# Quality and outcomes of maternal and perinatal care for 76,563 pregnancies reported in a nationwide network of Nigerian referral-level hospitals

**DOI:** 10.1016/j.eclinm.2022.101411

**Published:** 2022-04-28

**Authors:** Jamilu Tukur, Tina Lavin, Abiodun Adanikin, Muhammed Abdussalam, Kuti Bankole, Mabel Ikpim Ekott, Akaba Godwin, Halima A Ibrahim, Okonkwo Ikechukwu, Saidu Abubakar Kadas, Linda Nwokeji-Onwe, Emily Nzeribe, Taofik Oluwaseun Ogunkunle, Lawal Oyeneyin, Karima A. Tunau, Musa Bello, Is'haq Aminu, Bosede Ezekwe, Peter Aboyeji, Olubukola A. Adesina, Calvin Chama, Saturday Etuk, Hadiza Galadanci, Joseph Ikechebelu, Olufemi T. Oladapo, Maternal and Perinatal Database for Quality, Equity and Dignity Network, Abiodun S Adeniran, Aishatu A Gobir, Amaka Ocheke, Fatimah Baba Joy, Ibrahim Rais, Amsa B Mairami, Mohammed S. Ozegya, Samuel Pam, Sarah Ango, Musa Abdulkarim Omoyine, Medupin Patricia, Silas Ochejele, Egwu Agada, Duum Nwachukwu, Grace Ahmed, Aisha Abdurrahman, Lawal M Ibrahim, Aisha Nana Adamu, Aliyu Na'uzo, Adewale Ashimi, Umma Idris, Owodunni A Adebola, Festus D Akeredolu, Asma'u Adamu, Aliyu Labaran, Adekunle Oguntayo, Abdulkadir Isa, Stephen Bature, Andeyantso E Ayuba, Hauwa Abdullahi, Zubaida L Farouk, Sulaiman Muhammad Daneji, Umar Isa, Samuel Adelaiye, Ismail M Kalle, Saidu A Kadas, Muhammad F Bashir, Joel Moruppa, Wasinda S Bulus, Usman R. Yahaya, Jalo Iliya, Abdulkarim Mairiga, Adamu Atterwahmie, Abdulhakeem Hamza, Ishaya Wanonyi, Uniga A John, Wole Ayegbusi, Adefemi Ayodeji, Zainab Imam, Opeyemi Akinajo, Iretiola Fajolu, Olufemi Akinsanya, Efeturi Agelebe, Timothy Oluwasola, Olukemi O Tongo, Olusoji Jagun, Kuponiyi Opeyemi, Olumide Kuku, Abimbola Akindolire, David O Awonuga, Iyabode Olabisi F. Dedeke, Francis Akinkunmi, Babatunde Olofinbiyi, Ogundare E Olatunde, Olufemi Aworinde, Efeturi Agelebe, Olusoji Adeyanju, Campbell Ibijoke, Adedapo B Ande, Aniekan Abbasiatai, Eno Etim Nyong, Sunny Ochigbo, Lawrence Omo‐Aghoja, Patrick Ekpebe, Anthonia Njoku, Andrew Eigbedion, Ngozi Orazulike, Chioma Okechukwu, Solomon Igbaruma, Idemudia Ebe, Osahon Ede-Edokpolor, Amarabia Ibeawuchi, Isa Ayuba Ibrahim, Oyedeji O Adeyemi, Chukwuemeka C Mgbafulu, Onubogu C Ukamaka, Ugwu Anayochukwu, Uchenna Ekwochi, Obinna-Njoku Chioma, George Eleje, Eziamaka P Ezenkwele, Ijeoma Obumneme-Anyim, Nnabuike Ojiegbe, Nathan U Nwokeforo, Ifeanyichukwu Ezebialu, Obiora Ejiofor

**Affiliations:** a Department of Obstetrics and Gynaecology, Aminu Kano Teaching Hospital, Kano, Nigeria; b UNDP/UNFPA/UNICEF/WHO/World Bank Special Programme of Research, Development and Research Training in Human Reproduction (HRP), Department of Sexual and Reproductive Health and Research, World Health Organization, Geneva, Switzerland; c Department of Paediatrics, Aminu Kano Teaching Hospital, Kano, Nigeria; d Department of Paediatrics, Obafemi Awolowo University Teaching Hospital Complex, Ile-Ife, Nigeria; e Department of Obstetrics and Gynaecology, University of Calabar Teaching Hospital, Calabar, Nigeria; f Department of Obstetrics and Gynaecology, University of Abuja Teaching Hospital, Gwagwalada, Nigeria; g Department of Paediatrics, University of Maiduguri Teaching Hospital, Maiduguri, Nigeria; h Department of Paediatrics, University of Benin Teaching Hospital, Benin City, Nigeria; i Department of Obstetrics and Gynaecology, Abubakar Tafawa Balewa University Teaching Hospital, Bauchi, Nigeria; j Department of Paediatrics, Alex Ekwueme Federal University Teaching Hospital, Federal Teaching, Abakaliki, Nigeria; k Department of Obstetrics and Gynaecology, Federal Medical Centre, Owerri, Nigeria; l Department of Paediatrics, Dalhatu Arab Specialist Hospital, Lafia, Nigeria; m Department of Obstetrics and Gynaecology, University of Medical Science Teaching Hospital, Ondo, Nigeria; n Department of Obstetrics and Gynaecology, Usman Dan Fodiyo University Teaching Hospital, Sokoto, Nigeria; o Department of Community Medicine, Aminu Kano Teaching Hospital, Kano, Nigeria; p Data Analysis Unit, National Coordinating Secretariat, Maternal and Perinatal Database for Quality, Equity, and Dignity Programme, AKTH, Kano, Nigeria; q Department of Ageing and Life Course, World Health Organization, Nigeria Country Office, Abuja, Nigeria; r Department of Obstetrics and Gynaecology, University of Ilorin Teaching Hospital, Ilorin, Nigeria; s Department of Obstetrics and Gynaecology, University College Hospital, Ibadan, Nigeria; t Department of Obstetrics and Gynaecology, Abubakar Tafawa Balewa University Teaching Hospital, Bauchi, Nigeria; u Department of Obstetrics and Gynaecology, University of Calabar Teaching Hospital, Calabar, Nigeria; v African Center of Excellence for Population Health and Policy, Bayero University Kano, Nigeria; w Department of Obstetrics and Gynaecology, Nnamdi Azikiwe University Teaching Hospital, Nnewi, Nigeria

**Keywords:** Maternal mortality, Neonatal mortality, Maternal morbidity, Quality of care

## Abstract

**Background:**

The WHO in collaboration with the Nigeria Federal Ministry of Health, established a nationwide electronic data platform across referral-level hospitals. We report the burden of maternal, foetal and neonatal complications and quality and outcomes of care during the first year.

**Methods:**

Data were analysed from 76,563 women who were admitted for delivery or on account of complications within 42 days of delivery or termination of pregnancy from September 2019 to August 2020 across the 54 hospitals included in the Maternal and Perinatal Database for Quality, Equity and Dignity programme.

**Findings:**

Participating hospitals reported 69,055 live births, 4,498 stillbirths and 1,090 early neonatal deaths. 44,614 women (58·3%) had at least one pregnancy complication, out of which 6,618 women (8·6%) met our criteria for potentially life-threatening complications, and 940 women (1·2%) died. Leading causes of maternal death were eclampsia (*n* = 187,20·6%), postpartum haemorrhage (PPH) (*n* = 103,11·4%), and sepsis (*n* = 99,10·8%). Antepartum hypoxia (*n* = 1455,31·1%) and acute intrapartum events (*n* = 913,19·6%) were the leading causes of perinatal death. Predictors of maternal and perinatal death were similar: low maternal education, lack of antenatal care, referral from other facility, previous caesarean section, latent-phase labour admission, operative vaginal birth, non-use of a labour monitoring tool, no labour companion, and non-use of uterotonic for PPH prevention.

**Interpretation:**

This nationwide programme for routine data aggregation shows that maternal and perinatal mortality reduction strategies in Nigeria require a multisectoral approach. Several lives could be saved in the short term by addressing key predictors of death, including gaps in the coverage of internationally recommended interventions such as companionship in labour and use of labour monitoring tool.

**Funding:**

This work was funded by MSD for Mothers; and UNDP/UNFPA/ UNICEF/WHO/World Bank Special Programme of Research, Development and Research Training in Human Reproduction (HRP), a co-sponsored programme executed by the World Health Organization (WHO).

**Research in context**

**Evidence before this study**

There are few examples of successful national level perinatal database programmes in sub-Saharan Africa to support quality improvement strategies. The first attempt to establish a nationwide perinatal data system in Nigeria, the leading contributor to the global burden of maternal death, was through a research platform that collected data on maternal deaths and near-misses between 2012 and 2013. While this platform made important contributions regarding the causes of severe maternal complications in Nigerian tertiary hospitals, the lack of individual-level data on women without complications (who could have served as controls) prevented a comprehensive understanding of the factors associated with the observed suboptimal quality of care.

**Added value of this study**

The large number of women and neonates and high burden of mortality in this cohort allowed for identification of several independent predictors of maternal and perinatal death. The most striking findings were the increased odds of maternal death, and of perinatal death when there was no labour companion. Another important finding was the strong association between maternal death and non-use of a labour monitoring tool. These findings have not been reported in randomized trials or systematic reviews of these interventions probably due to the prohibitively large samples sizes and high event rates that would be needed to detect such important differences between comparison groups. To our knowledge, this is the first study demonstrating an association between these globally recommended interventions and maternal and perinatal mortality.

**Implications of all the available evidence**

Prospective surveillance of maternal and perinatal data, including the use of selected quality of care indicators, for periodic assessment of hospital performance and quality improvement is critical for achieving the aims of the WHO QED initiative and for meeting the third sustainable development goal targets at the country level. A nationwide database programme for harmonizing and aggregating data could be implemented in settings with similar medical record infrastructure as Nigeria, to identify interventions that could be readily implemented to drive policy change and impact. With the global shift towards increased facility births and the digitization of routine national health management systems, there is a huge potential to scale up this programme to other countries.  Our study identified important gaps in the coverage of several interventions, such as labour companion which could readily be addressed in the short term to improve maternal and newborn survival at the highest level of health care delivery in Nigeria and other countries with similar hospital settings.

Alt-text: Unlabelled box

## Introduction

Global efforts to reduce maternal and perinatal deaths are targeted at reducing the global maternal mortality ratio to less than 70 per 100,000 live births and neonatal mortality rate to less than 12 per 1000 live births by 2030 through the sustainable development agenda.^1^ In 2017, Nigeria had a maternal mortality ratio of 512 per 100,000 live births, the highest in Africa, and in 2015 neonatal mortality was 38 per 1000 live births, second only to India.^2^^,^^3^ The lack of reliable maternal and perinatal data in Nigeria for health care planning has remained a challenge for programme managers, health care advocates and policy makers, and thus impedes progress towards reaching the global targets.

Challenges with measurement of the burden of maternal and perinatal mortality and morbidity, and quality of care in low- and middle-income countries (LMICs) are common. Data systems are often paper-based and not centralized for aggregation at the national level. There are few examples of successful national level perinatal database programmes in sub-Saharan Africa to support quality improvement strategies. These few examples, such as the Perinatal Problem Identification Program (PPIP) in South Africa and Lesotho aggregate facility-level data, and therefore are limited in their capacity to report detailed information to support broader quality improvement strategies beyond mortality reduction.^4^

The first nationwide maternal data system in Nigeria was through a research platform, the Nigeria Near-miss and Maternal Death Survey, that collected data on 998 maternal deaths and 1451 near-misses between 2012 and 2013.^5^ The multi-centre cross-sectional study, set in 42 public tertiary hospitals providing obstetric services within the six geopolitical zones of Nigeria, identified women who died or experienced a maternal near-miss from pregnancy, childbirth or puerperal complications based on uniform identification criteria. While this study revealed important information on the burden, causes and avoidable factors contributing to severe maternal complications, a comprehensive understanding of the factors associated with the observed suboptimal quality of care was not possible due to the lack of individual-level data on women without complications (who could have served as controls). Further, the potential gains from policy changes in Nigeria after the publication of this landmark study could not be sustained, as the platform was not continued beyond the research project.

The aim of the current study was to address the gap in the availability of harmonized nationwide maternal and perinatal quality of care data (including data for healthy pregnant women) in tertiary level facilities across Nigeria to examine the burden and causes of maternal, foetal, and early neonatal complications, the factors associated with death, and indicators of quality of care.

This study was established as part of the World Health Organization's (WHO) Quality, Equity and Dignity (“QED”) programme in nine countries (including Nigeria) with the aim of halving intrahospital maternal and neonatal deaths in five years. One of the quality of care standards was that every mother and newborn has a complete, accurate, standardized medical record during labour, childbirth and the early postnatal period. For the WHO QED vision to be realised, it was expected that every health facility has a mechanism for routine data collection, analysis and feedback as part of its activities for monitoring and improving performance around the perinatal period.

WHO, in collaboration with the Nigeria Federal Ministry of Health, established a nationwide electronic data platform across a network of referral-level hospitals to collect routine data during labour, childbirth, and early postnatal period. The platform harmonized the assessment of quality of care provided to women and newborns around the time of birth, and standardized audits of maternal and perinatal deaths at the tertiary level of health care delivery. The platform also enabled data collection on selected quality indicators that were proposed for the WHO QED countries – the ‘QED indicators’.

Here we report the burden and spectrum of maternal, foetal, and early neonatal complications, causes and predictors of maternal and perinatal death, and indicators of quality of care during the first year of setting up this platform. This study was conducted to provide insight into the burden of severe complications around the time of childbirth, the quality of maternal and perinatal care, and drivers of maternal and perinatal deaths in Nigerian referral-level facilities.

## Methods

### Study design, sampling and participants

This cross-sectional study captured maternal and perinatal data in a nationwide network of 54 consenting tertiary hospitals, serving as referral centres for other health facilities in their environs (48 publicly funded and 6 privately funded), across the six geopolitical zones of Nigeria (Northcentral, Northeast, Northwest, Southeast, Southsouth, Southwest). All (*n* = 52) publicly funded referral-level hospitals providing in-patient services for obstetric and gynaecological admissions were targeted for inclusion in the study. Of these, 48 hospitals (92.4%) provided consent, participated and successfully implemented the study. In addition two referral-level privately funded facilities in each region were targeted for inclusion. Six privately funded hospitals located in Northwest (*n* = 2), Southwest (*n* = 2), and Southsouth (*n* = 2) regions consented and participated in the study.

The study population comprised of all women (and their babies) who were admitted for delivery or on account of complications within 42 days of delivery or termination of pregnancy between 1 September 2019 and 31 August 2020. This population was chosen to account for all pregnancy-related complications that could result in severe morbidity or death in the participating hospitals as well as the standard denominators for estimating the burden of maternal, foetal, and neonatal mortality (live births and all births). Additionally, women who experienced a pregnancy loss or had an abortion were included, an often neglected population in national maternal and/or perinatal health databases. The 42-day time period for including women after giving birth or termination of a pregnancy ensured that all women whose deaths could be classified as a maternal death were enrolled. For the purpose of data entry, a woman was categorised as an obstetric admission if she was admitted for delivery at or after 28 weeks of gestation or if she was admitted within 42 days following delivery. A woman was categorised as a gynaecological admission if she was admitted with a pregnancy (<28 weeks) that ended in spontaneous, induced or missed abortion, molar pregnancy, ectopic pregnancy, or intrauterine foetal death.

The scientific content of the study was approved by the WHO Human Reproduction Programme (HRP) Research Project Review Panel (protocol ID, A65930, 06 May 2018). WHO Ethics Review Committee (ID A65930, 05 June 2018) and the Nigerian National Health Research and Ethics Committee approved the study (ID NHREC/01/01/2007, 05 September 2018). Authorities of all participating hospitals granted written institutional approvals to participate in the programme's data collection, periodic analyses and reporting. Individual level written consent was not required as the study did not involve direct interaction with women or their babies, or interview of medical staff.

### Development of the platform and programme coordination

The electronic data platform was developed by customising open-source District Health Information Software (DHIS-2), the approved Health Management Information System by the Federal Ministry of Health of Nigeria. The data platform collected information from patient medical records using an electronic case report form. The information obtained included women's sociodemographic data, past medical history, antenatal history, labour and delivery details (including the baby's clinical condition), and immediate postpartum observations. The data collected were used to report on the QED indicators specified for the multinational WHO QED initiative (Appendix II).^6^

There was a National Coordinating Unit, comprising of a national coordinator, a data manager, a statistician, six regional coordinators (each overseeing facilities in their region), Nigeria Ministry of Health representatives and WHO staff from the Nigeria country office and headquarters. Each of the participating hospitals had a team of one obstetrician, one neonatologist (designated as hospital coordinators) and two medical record officers (MROs) coordinating and entering data from patient medical records into the database. Each hospital had quarterly facility audits conducted by their hospital coordinators on available services, human resources and supplies.

### Data collection and electronic capture

Trained MROs (two at each hospital) conducted daily surveillance of medical records in the obstetric ward, gynaecological emergency unit, birthing/delivery room, operating theatre and intensive care unit. For each woman, data entry was initiated at time of admission, updated during admission, and completed at time of discharge or death (whichever was earlier). Data were captured from patient medical records using a tablet-based case report form that was specifically developed for the project. A unique identifier was used to link the data between a woman and her newborn in the database.

In the event of a maternal or perinatal death (stillbirth or early neonatal death), the local mortality audit team (led by an obstetrician and neonatologist) analysed and documented the primary cause of death (using International Classification of Diseases for maternal death [ICD-MM] and perinatal death [ICD-PM]),^7^^,^^8^ and the associated avoidable factors.^9^ In cases of re-admission of a woman (within 42 days of delivery) or neonate (within the first 7 days of life), a re-admission form was generated to record the reason(s) for the re-admission and the outcome. A re-admission was not considered a new entry and additional information from the re-admission was added to the previously recorded data for the woman or neonate.

The data collected were synchronised in real-time from the internet-enabled tablet device to a secured central cloud-base server. The data record for each participant was closed 60 days after admission – allowing for correction of any data entry error by hospital coordinators, generation of a re-admission form (in case of a re-admission), and completion of maternal or perinatal death audit form where applicable.

To ensure reliability of data and minimise heterogeneity in data collection across hospitals, several quality assurance procedures were put in place. A manual of standard operating procedures was developed and training provided to project teams from all facilities. The data platform had in-built validation rules, including the use of mandatory fields, to minimise data entry errors and ensure completeness of data. Before synchronising with the central server, data entered for each woman and her baby were verified by the hospital coordinators. The data manager at the National Coordinating Unit and a staff at the WHO Country Office conducted monthly checks of total admissions in relevant hospital registers against the admissions recorded in the database, and took necessary actions to address any discrepancies. In addition, approximately 5% of woman-infant records were randomly selected and scrutinised for any errors or inconsistencies on a weekly basis by the hospital coordinator. Any inconsistencies or errors identified were resolved before closure of the data record for each participant, ensuring only accurate data were included in the database.

### Outcomes and definitions

The availability of live-saving interventions (blood banking services, neonatal resuscitation facilities, operating theatre and anaesthetic machine), and availability of oxytocin, ergometrine, tranexamic acid, magnesium sulfate, intravenous fluids, and strength of the health care workforce were assessed using data obtained from quarterly facility-level audits.

Key outcomes for women and their babies included the burden and causes of morbidity and mortality, avoidable factors contributing to mortality, and independent predictors of mortality. We assessed the burden of maternal morbidity according to the morbidity continuum adapted from Say et al.^10^ with the following categories: women with any complication regardless of severity, women with potentially life-threatening complication, women who survived life-threatening complication (severe acute maternal morbidity), and maternal death. The full description of these categories can be found in Appendix III.

The burden of maternal mortality was determined by calculating the intrahospital maternal mortality ratio (defined as number of maternal deaths amongst all women admitted and managed in the hospital regardless where they gave birth, per 100,000 live births); and pre-discharge maternal mortality ratio (defined as number of deaths amongst women who delivered in the hospital prior to discharge per 100,000 hospital live births). While the former was based on standard definition of maternal mortality ratio at the hospital level, the latter had been specifically developed as one of the QED indicators.

The burden of perinatal mortality was estimated by calculating the QED indicators institutional stillbirth rate and pre-discharge neonatal mortality rate. Institutional stillbirth rate was disaggregated by antenatal and intrapartum stillbirths and was defined as the number of babies born in a health facility with no signs of life at birth, per 1000 facility births, and pre-discharge neonatal mortality rate was defined as the number babies born live in a facility who died prior to discharge per 1000 facility live-births.

Avoidable factors contributing to maternal and perinatal deaths were assigned by the hospital mortality audit team using the National Maternal and Perinatal Death Audit Tool that was developed and published by the Nigerian Federal Ministry of Health.^9^ Avoidable factors included patient-orientated and facility-level factors as defined in the National Maternal and Perinatal Death Audit Tool (Appendix IV). Avoidable factors experienced by women and their babies prior to their arrival at the hospital and upon arrival at hospital were based on evaluation of patient medical records and were ascertained by the hospital mortality audit team.

The quality of care provided in the participating hospitals was assessed using a list of indicators that were developed by a consultative group of experts to standardize quality assessment across the nine countries participating in the WHO QED initiative.^6^ The definitions and measurement of these QED indicators are presented in Appendix II. To complement this assessment, the overall quality of care performance for specific maternal conditions were also assessed using cause-specific case fatality rates. Cause-specific case fatality rate was defined as the proportion of women who died from a specific condition amongst all women with the condition. Cause-specific case fatality rates were calculated at the national and regional levels.

The following sociodemographic and clinical variables were separately analysed as independent predictors of maternal and perinatal deaths: maternal age, marital status, highest education level attained, woman's occupation, husband/partner's occupation, previous miscarriage(s), previous caesarean section(s), parity, antenatal care, referral status, cervical dilatation at admission, mode of birth, companionship in labour (for obstetric admissions), continuous labour monitoring with a partograph (in women who underwent labour) and use of uterotonic for the prevention of postpartum haemorrhage.

### Statistical analysis

We conducted descriptive analysis on the available human resources and supplies at the facility level, as well as the sociodemographic and clinical characteristics of the study population at the individual level. We performed descriptive analysis of the proportion of women in each category of the maternal morbidity continuum. We calculated intrahospital maternal mortality ratio and pre-discharge maternal mortality ratio, the live births and stillbirths (by place of birth), based on the above definitions, for all facilities, and for publicly-funded and privately-funded facilities separately. Descriptive statistics were used to examine the causes of maternal and perinatal deaths and avoidable factors contributing to deaths.

Independent predictors for maternal death, and for perinatal death were separately explored using logistic regression models. Each variable was first entered in a univariable logistic regression model with death as the binary outcome for unadjusted estimates. Variable levels were aggregated where appropriate. Multilevel mixed-effects logistic regression models were used to determine the sociodemographic and clinical characteristics that were associated with maternal death. A similar model was used to determine the variables associated with perinatal death. Random effects were adjusted for at the hospital level. A backward stepwise variable selection procedure was used to progressively remove the least useful predictors until the most parsimonious model was achieved. Unadjusted and adjusted odds ratios (OR) with corresponding 95% confidence intervals (CI) and p-values are reported. Statistical significance was accepted at *p*<0·05. All analyses were conducted with STATA Version 16 (College Station, TX: StataCorp LLC).^11^

Variables included in the univariable and multivariable models with >5% missing data were checked for type of missingness (missing not at random, missing completely at random, missing at random).^12^ Where necessary, sensitivity analysis was conducted by comparing complete case analyses with models including missing data. The use of multiple imputation for missing values was explored where appropriate.

Role of funding source: The funders did not play any role in the study design; in the collection, analysis, and interpretation of data; in the writing of the report; and in the decision to submit the paper for publication. JT, TL, MB, OO had access to the data. The decision to submit the paper for publication was taken by all authors.

## Results

### Implementation of the programme

The key features of the Maternal and Perinatal Database for Quality, Equity and Dignity Programme are presented in Table S1.

### Characteristics of participating hospitals

Facility-level audit of 46 participating hospitals (1 privately-funded and 7 publicly-funded did not complete audits) showed that life-saving medications for prevention and treatment of postpartum haemorrhage and eclampsia were consistently available in the majority of the hospitals: oxytocin (46 facilities, 100%), intravenous fluids (46 facilities, 100%), ergometrine (42 facilities, 91·6%), and magnesium sulfate (44 facilities, 95·8%). The exception was tranexamic acid which was consistently available in 26 of the hospitals (56.3%). The available human resources, number of women admitted, and foetal, maternal, and early neonatal mortality outcomes reported during the study period are presented in Table S2. There was no clear relationship between the number of obstetric and neonatal care providers employed in the participating hospitals and the mortality outcomes reported during the study period.

### Characteristics of study population

Between 1 September 2019 and 31 August 2020, a total of 76,563 women were admitted for delivery or on account of complications within 42 days of delivery or termination of pregnancy. 4079 of these women (5.3%) delivered at a privately-funded facility. There were 74,385 babies born at or above 28 weeks to the women in this study. The sociodemographic and clinical characteristics of the women and their babies are presented in Table 1.

**Table 1:** Sociodemographic and clinical care characteristics of women admitted for delivery or on account of complications within 42 days of delivery or termination of pregnancy.

Characteristic ^+^	All women (*n* = 76,563)
	n (%)
Age (years, mean, SD)	29.4 (5.8)
*Missing*	522 (0.7%)
Marital status	
Single	1215 (1.6)
Married/cohabitating	74,447 (97.2)
Separated/divorced	113 (0.2)
Widowed	13 (0.02)
*Missing*	775 (1.0)
Education level (highest level attained)	
No formal education	7685 (10.1)
Primary education	2812 (3.7)
Secondary education	29,170 (38.1)
Post-secondary education	29,905 (39.2)
*Missing*	6039 (7.9)
Occupation – woman	
Not gainfully employed	28,196 (36.8)
Professional/technical/managerial	5051 (668)
Civil servant/administrative/clerical	12,318 (16.0)
Sales/trading	17,225 (22.5)
Farming/agricultural work/skilled manual/unskilled manual	5494 (7.2)
Other	3312 (4.3)
Unknown	2642 (3.5)
*Missing*	2325 (3.0)
Occupation – husband/partner	
Not gainfully employed	860 (1.1)
Professional/technical/managerial	6920 (9.0)
Civil servant/administrative/clerical	24,123 (31.5)
Sales/trading	18, 865 (24.6)
Farming/agricultural work/skilled manual/unskilled manual	10,454 (13.7)
Other	3168 (4.1)
Unknown	11,130 (14.5)
*Missing*	1043 (1.4)
Tribe	
Hausa	18,839 (24.6)
Yoruba	16,155 (21.1)
Igbo	12,840 (16.8)
Fulani	3904 (5.1)
Kanuri	1849 (2.4)
Tiv	1783 (2.3)
Binis	1573 (2.1)
Esan	1230 (1.6)
Other	17, 078 (22.3)
*Missing*	1312 (1.7)
Religion	
Christianity	40,203 (52.5)
Islam	35,012 (45.7)
Traditional	106 (0.1)
Other	59 (0.08)
*Missing*	1183 (1.5)
Booking status	
No antenatal care (ANC)	11,283 (14.7)
ANC at the same facility	51,930 (67.2)
ANC at another health facility	11,613 (15.2)
ANC outside of a health facility	352 (0.5)
*Missing*	1385 (1.8)
Median number ANC visits for women who had ANC at same facility	6
*Missing*	3332 (7.8)
Parity	
First pregnancy	22,919 (29.9)
1–4 previous pregnancies (≥ 28 weeks)	46,193 (60.3)
5 or more previous ≥28 weeks	6920 (9.1)
*Missing*	531 (0.7)
Number previous miscarriages	
0	54,129 (70.7)
1	12,232 (15.9)
2	5229 (6.8)
3	1956 (2.6)
4 or more	1209 (1.6)
*Missing*	1808 (2.4)
Number previous caesarean section	
0	63,630 (83.1)
1	6626 (8.6)
2	2679 (3.4)
3 or more	763 (1.0)
*Missing*	2865 (3.7)
Source of referral for women referred to hospital	
Self-referral	3980 (26.3)
Public hospital	7932 (52.4)
Private hospital	2586 (17.1)
Mission home	165 (1.1)
Traditional Birth Attendant	184 (1.2)
Other	293 (1.9)
*Missing*	3 (0.0)
Location where women gave birth	
Same health facility (within the network)	69,799 (97.2)
Other health facility	545 (0.7)
Non-health facility	97 (0.1)
Home	546 (0.8)
Other location	165 (0.2)
*Missing*	606 (0.8)
Cervix dilatation at admission	
Latent phase (0–3 cm)	44,398 (61.9)
Active phase (4 or above)	27,372 (38.1)
*Missing*	0 (0)

+ Number of women varies for characteristics between obstetric and gynaecological admissions.

### Care during labour and childbirth

#### Use of labour monitoring tool

There were 54,923 women who underwent labour. Of these, 43,171 (78·6%) had continuous labour monitoring with a partograph, 9316 (16.9%) did not have continuous labour monitoring with partograph and it was unknown if 1684 (3.1%) women had continuous labour monitoring with partograph. There were missing data for 752 women (1.4%).

#### Companionship

Of obstetric admissions, 18,944 women (26.4%) had a companion in labour, 35,341 women (49.2%) had no companion in labour and for 15,098 women (21.0%) it was not known whether a companion was present. Data were missing for 2375 women (3.3%). Of the 18,944 women who had a companion during labour, 55.2% had their spouse, 38.5% had a family member and 6.3% had a person who was not a family member. Women with a companion in labour had more spontaneous vaginal deliveries compared to women without a companion (Risk Difference (RD) 12 more per 1000, 95% CI 4 more to 21 more), fewer caesarean sections (RD 10 more per 1000, 95% CI 2 fewer to 18 fewer) and fewer instrumental vaginal births (RD 3 fewer per 1000, 95% CI 1 fewer to 4 fewer). Women with a companion in labour also had fewer babies with 5 min Apgar scores less than 7 (9 fewer per 1000, 95%CI 1 fewer to 5 fewer).

#### Uterotonic use for post-partum haemorrhage prevention

There were 58,809 (81.9%) women who received a uterotonic for PPH prevention, 6241 (8.7%) who did not receive a uterotonic for PPH prevention and it was unknown if 5972 (8.3%) women received a uterotonic for PPH prevention. There were missing data on 748 (1.1%) of women.

### QED indicators

Table 2 presents the QED indicators that were explored across pregnancies in the network.

**Table 2:** *Quality, Equity, and Dignity* (QED) Indicators findings.

QED indicator	National	Range across regions
Pre-discharge maternal mortality ratio ^§^	682 per 100,000 hospital live births	430 per 100,000 to 974 per 100,000
Institutional stillbirth rate	59·3 per 1000 hospital births	40.1 per 1000 hospital births to 90.1 per 1000
Antenatal stillbirth rate	26·8 per 1000 hospital births	18.9 per 1000 to 34.2 per 1000
Intrapartum stillbirth rate	32·3 per 1000 hospital births	21.2 per 1000 to 56.0 per 1000
Pre-discharge neonatal mortality rate	15·3 per 1000 hospital live births	13.3 per 1000 to 20.2 per 1000
Pre-discharge family planning counselling for mother and baby	74·8%	64·5% to 86·9%
Companion in labour	26·4%	17·1% to 35·1%
Newborns breastfed within one hour of birth	59·7%	52·8% to 67·1%
Immediate postpartum uterotonic use for postpartum haemorrhage (PPH) prevention	81·9%	70·5% to 94·2%
Newborns with birthweight documented	96·6%	91·1% to 97·9%
Basic hygiene provision *	91·0%	41·0% to 100%
Basic sanitation for women and families *	89·0%	43·0% to 100%

^§^832 births have missing data on vital status at birth these have been excluded from mortality calculations; * facility level indicators.

### Burden of maternal and perinatal complications

**Figure. 1:**
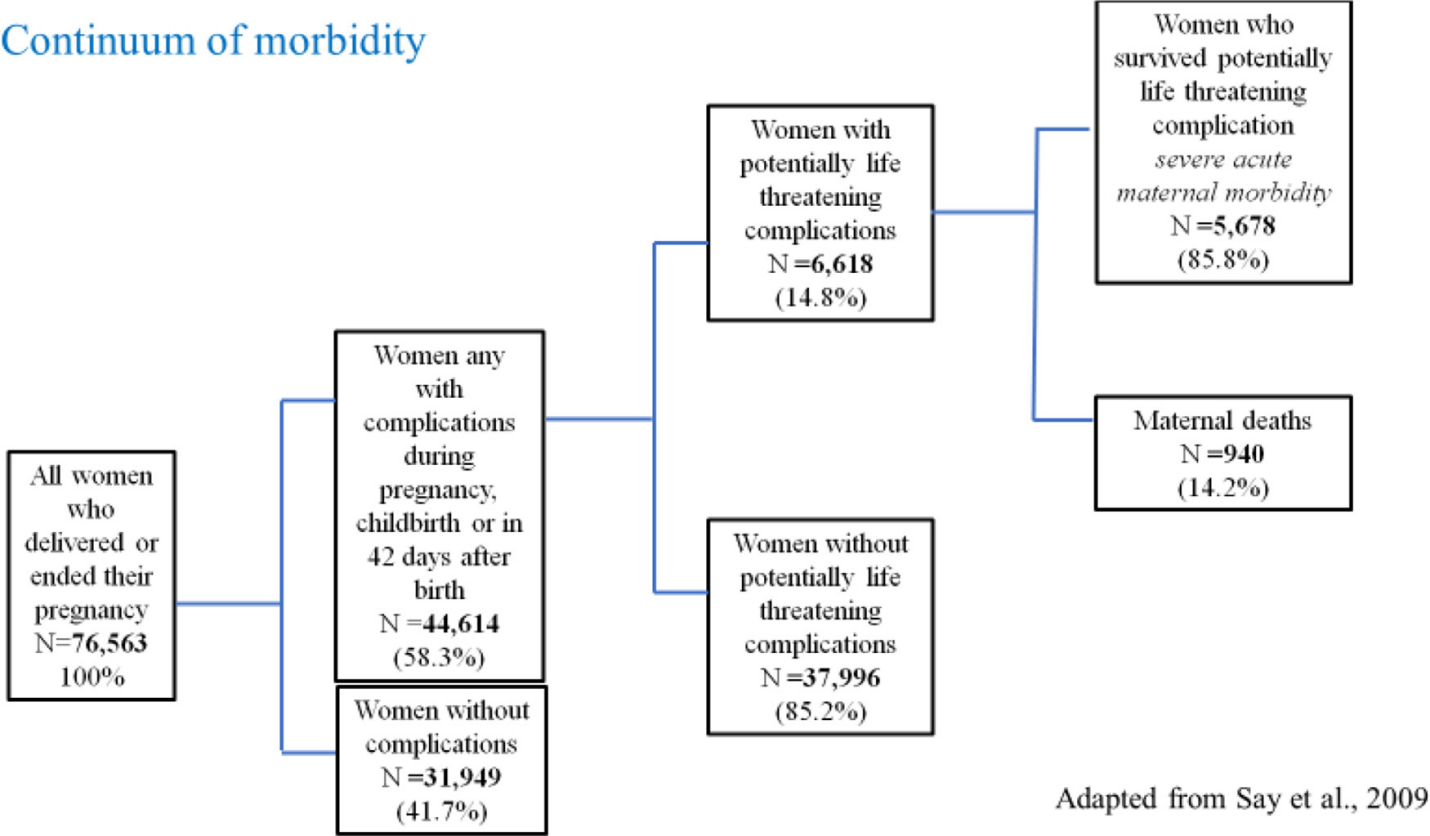
Morbidity continuum for all women who were admitted to participating hospitals for delivery or on account of complications within 42 days of delivery or termination of pregnancy. Percentages to proportion of women at each stage of morbidity continuum (not total number of women).

37,998 women (49.6%) admitted during pregnancy, childbirth or 42 days after childbirth or termination of pregnancy had a complication that was not life-threatening, 5678 (7·4%) had a potentially life threatening complication but survived, and 940 (1·2%) of women died. 1.3% (931/72,484) of women who delivered in publicly-funded facilities died, and 0.2% (9/3857) of women who delivered in privately-funded facilities died. The morbidity continuum is presented in Figure. 1.

Intrahospital maternal mortality ratio was 1361 per 100,000 live births (940/69,055) and pre-discharge maternal mortality ratio was 682 per 100,000 hospital live births (464/67,971). Maternal mortality rates were lower for women who delivered in privately-funded facilities (209 per 100,000; 8/3819) compared to publicly-funded (710 per 100,000; 456/64,152). 526 (56%) of maternal deaths were women who had been referred. Maternal death audits were completed in 909 (96·7%) of cases. The primary causes of maternal death are presented in Table 3. The leading causes of maternal death were hypertensive disorders of pregnancy (*n* = 289, 31·8%), pregnancy-related infections (*n* = 146, 16·1%), and postpartum haemorrhage (*n* = 103, 11·4%). Cause-specific case fatality rates were worst for puerperal sepsis (40·8%) and eclampsia (24·9%) (Table S3).

**Table 3:** Primary cause of maternal death using the WHO application of ICD-10 to maternal mortality (ICD-MM) (5).

Primary cause of death ^+^	*N* = 909 (%)
Obstetric haemorrhage	181 (19.9)
Placental disorders	3 (0.3)
Placenta praevia	14 (1.5)
Premature separation of placenta [abruptio placentae]	33 (3.6)
Antepartum haemorrhage, not elsewhere classified	5 (0.6)
Intrapartum haemorrhage	23 (2.5)
Postpartum haemorrhage	103 (11.4)
Pregnancy-related infection	146 (16.1)
Infections of genitourinary tract in pregnancy	7 (0.8)
Puerperal sepsis	98 (10.8)
Other puerperal infections	12 (1.3)
Other maternal infections and parasitic diseases	29 (3.2)
Abortive outcome	69 (7.6)
Haemorrhage in early pregnancy	4 (0.4)
Ectopic pregnancy	7 (0.8)
Hydatidiform mole	3 (0.3)
Other abnormal products of conception	7 (0.8)
Spontaneous abortion	8 (0.9)
Medical abortion	2 (0.2)
Other abortion	12 (1.3)
Unspecified abortion	11 (1.2)
Failed attempted abortion	5 (0.6)
Complications following abortion and ectopic and molar pregnancy	10 (1.1)
Hypertensive disorders	289 (31.8)
Pre-existing hypertensive disorder with superimposed proteinuria	19 (2.1)
Gestational [pregnancy-induced] hypertension without significant proteinuria	9 (0.9)
Gestational [pregnancy-induced] hypertension with significant proteinuria	61 (6.7)
Eclampsia	187 (20.6)
Unspecified maternal hypertension	8 (0.9)
Pre-existing hypertension complicating pregnancy, childbirth and the puerperium	5 (0.6)
Obstructed labour	22 (2.4)
Obstructed labour due to malposition and malpresentation of foetus	13 (1.4)
Other obstructed labour	9 (1.0)
Other direct obstetric complications	70 (7.7)
Embolism	12 (1.3)
Other obstetric trauma	14 (1.5)
Sequelae of complication of pregnancy, childbirth and the puerperium	29 (3.3)
Other direct causes	15 (1.6)
Indirect causes	63 (6.9)
Diabetes mellitus in pregnancy	8 (0.9)
Maternal disease (unspecified)	42 (4.6)
Complications of anaesthesia during pregnancy, labour and delivery	8 (0.9)
Other indirect complication	5 (0.5)
Other cause of death (unknown if direct or indirect cause)	51 (5.6)
Obstetric death of unspecified cause	18 (2.0)

^+^There were 940 deaths, with 909 maternal audits performed.

The most frequent avoidable factors contributing to maternal deaths were delay in woman seeking health care help (*n* = 641, 70·5%), delay in appropriate referral (*n* = 522, 57·4%) and lack/delay of transport from home to health facility (235, 25·8%) (Table 4).

**Table 4:** Avoidable factors contributing to maternal contributing factors to maternal death (*n* = 909).

Avoidable factor *	n (%)
Patient orientated factor	
Delay in woman seeking health care	641 (70.5)
Patient's refusal of treatment or admission	54 (5.9)
Facility level factor	
Delay in appropriate referral	522 (57.4)
Lack / delay of transport from home to health care facility	235 (25.8)
Lack of facilities, equipment or consumables	150 (16.5)
Delay in receiving care from medical staff	109 (12.0)
Health services and communication breakdown	90 (9.9)
Lack of medical expertise, training or education	76 (8.3)
Lack of human resources	46 (5.1)

*More than one factor can be selected for each death; 105 women did not have any contributing factor to death.

There were 74,385 babies born at or after 28 weeks of gestation. A total of 4498 of these babies were stillborn, and for 832 of them, the vital status at birth was unknown. Of the 69,055 live births, 1090 resulted in early neonatal death. The institutional stillbirth rate was 59·3 per 1000 facility births (4284/72,255). The stillbirth rate for publicly-funded facilities was 61.7 per 1000 (4217/68,369), and 17 per 1000 (67/3886) for privately-funded facilities. The antenatal stillbirth rate was 26·8 per 1000 facility births (1939/72,255) and the intrapartum stillbirth rate was 32·3 per 1000 facility births (2337/72,255)(for 8 stillbirths it was unknown if antenatal/intrapartum). The pre-discharge neonatal mortality rate was 15·3 per 1000 facility live-births (1043/67,971). The pre-discharge neonatal mortality rate was 15.8 per 1000 (1018/64,152) for publicly-funded facilities, and 6.5 per 1000 for privately-funded facilities (25/3819).

**Figure. 2:**
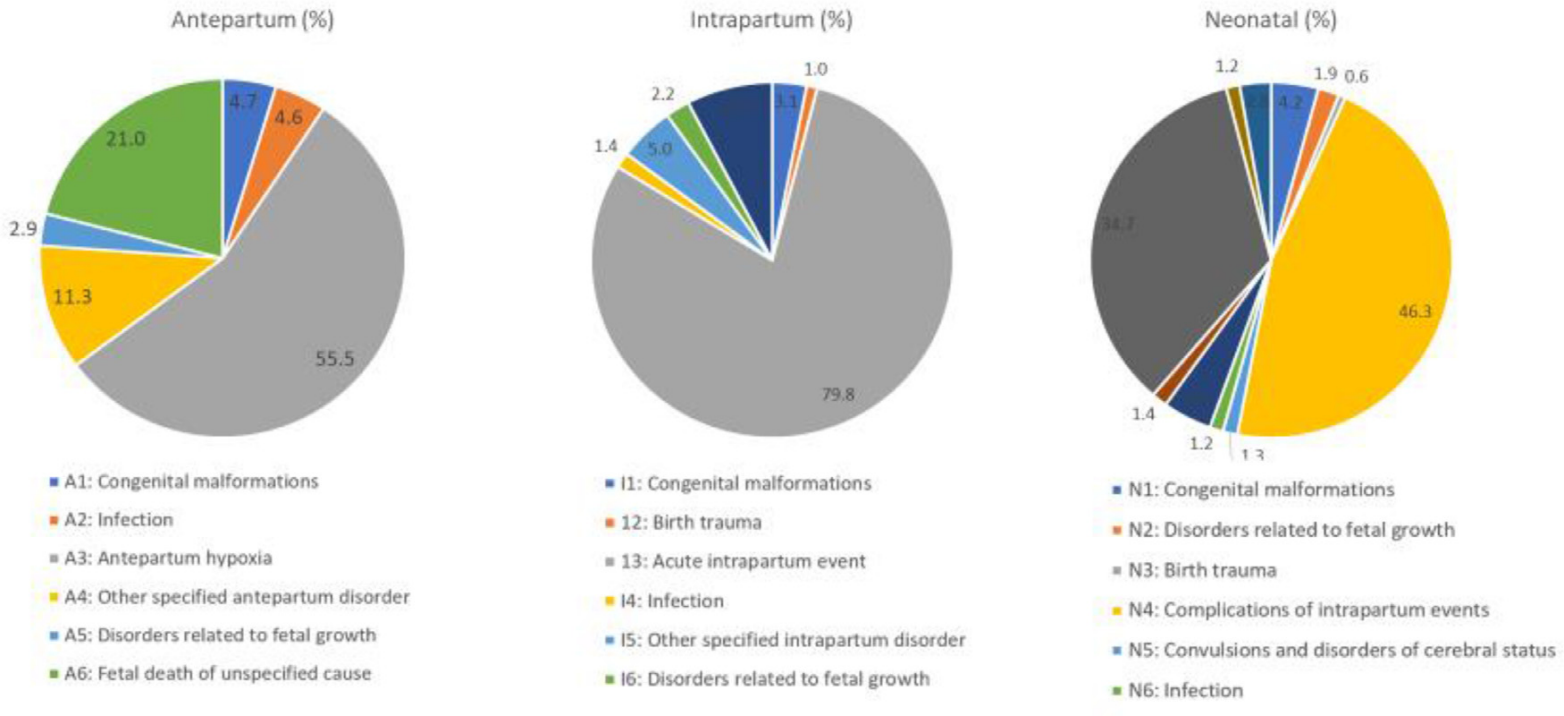
Causes of perinatal deaths using the WHO application of ICD-10 to perinatal mortality (ICD-PM) (*n* = 4,667).

Perinatal death audits were completed for 4667 deaths (87·6% of deaths occurring in facility). 2623 deaths occurred in the antepartum period (56·3%), 1144 occurred in the intrapartum period (24·5%) and 900 in the neonatal period (19·2%) (Figure 2). Most perinatal deaths occurred amongst women with a complication (*n* = 3539, 75·8%). The main causes of death are presented in Table S4. 2570 perinatal deaths were referrals (46%). 2576 (55.2%) of perinatal deaths had an avoidable family factor contributing to death, 1260 (27%) had an avoidable health worker factor, 980 (21%) had a community factor. Administrative factors were implicated in 313 (6·7%) of deaths.

### Independent predictors of maternal and perinatal death

Table 5 presents separately the predictors of maternal and perinatal death.

**Table 5:** Sociodemographic and clinical factors associated with maternal death ^±^ (*n* = 76,563) and perinatal death (*n* = 71,770)^§∞^, presented as odds ratio (OR) with 95% confidence intervals (95% CI).

Factors associated with death ª		Maternal death			Perinatal death		
		Unadjusted Odds Ratio (95% CI)	Adjusted Odds Ratio (95% CI)	p-value	Unadjusted Odds Ratio (95% CI)	Adjusted Odds Ratio (95% CI)	p-value
Age							
	<20 years	2.48 (1.94, 3.19)	1.17 (0.85, 1.61)	0.328	1.64 (1.48, 1.81)	0.86 (0.75, 0.99)	0.045
	*20–35 years (reference)*	1	1		1	1	
	>35 years	1.21 (1.01, 1.46)	1.15 (0.93, 1.44)	0.203	1.28 (1.21, 1.36)	1.26 (1.15, 1.37)	<0.001
Woman's education level							
	No formal education	8.44 (6.85, 10.39)	2.52 (1.86, 3.41)	<0.001	3.59 (3.37, 3.83)	1.68 (1.49, 1.89)	<0.001
	Primary education	6.71 (5.07, 8.87)	1.76 (1.24, 2.49)	0.002	2.96 (2.69, 3.26)	1.43 (1.24, 1.65)	<0.001
	Secondary education	2.13 (1.73, 2.62)	1.36 (1.07, 1.74)	0.012	1.52 (1.67, 1.96)	1.10 (1.01, 1.20)	0.022
	*Completed post-secondary education (reference)*	1	1		1	1	
Husband's occupation							
	Not gainfully employed	5.19 (3.11, 8.67)	2.09 (1.18, 3.67)	0.011	3.10 (2.63, 3.67)	1.48 (1.16, 1.88)	0.002
	*Professional/technical/managerial (reference)*	1	1		1	1	
	Sales/trading	2.78 (2.21, 3.48)	1.64 (1.27, 2.12)	<0.001	1.53 (1.45, 1.63)	1.29 (1.19, 1.42)	<0.001
	Manual labour/other	7.30 (5.89, 9.04)	1.98 (1.51, 2.59)	<0.001	2.99 (2.82, 3.18)	1.54 (1.39, 1.70)	<0.001
Antenatal care booking (ANC)							
	No antenatal care	15.1 (12.22, 18.66)	2.80 (2.10, 3.72)	<0.001	22.28 (21.01, 23.63)	5.58 (5.08, 6.14)	<0.001
	*ANC at the same facility (reference)*	1	1		1	1	
	ANC at another health facility	13.3 (10.76, 16.51)	2.09 (1.54, 2.82)	<0.001	7.97 (7.48, 8.48)	4.01 (3.62, 4.44)	<0.001
	ANC with traditional birth attendant or informal setting	28.8 (30.56, 52.09)	4.87 (2.75, 8.64)	<0.001	12.19 (9.70, 15.32)	5.00 (3.73, 6.70)	<0.001
Parity							
	First pregnancy	0.34 (0.28, 0.42)	0.91 (0.74, 1.12)	0.373	0.48 (0.45, 0.52)	0.97 (0.89, 1.04)	0.383
	*1–4 previous pregnancies (reference)*	1	1		1	1	
	5 or more previous	0.35 (0.29, 0.42)	1.34 (1.07, 1.69)	0.01	0.41 (0.38, 0.44)	1.59 (1.49, 1.75)	<0.001
Women has had previous caesarean section							
	*No (reference)*	1	1		1	1	
	Yes	0.88 (0.72, 1.1)	1.54 (1.18, 2.00)	0.001	0.57 (0.53, 0.61)	1.14 (1.03, 1.27)	0.016
Woman has had previous miscarriage							
	*No (reference)*	1	1		1	1	
	Yes	0.71 (0.59, 0.83)	0.76 (0.62, 0.92)	0.005	1.29 (1.23, 1.35)	1.18 (1.10, 1.27)	<0.001
Referral status							
	*Woman was not referred or self-referred (reference)*	1	1		1	1	
	Woman referred from public or private hospital	9.83 (8.50, 11.35)	4.03 (3.25, 4.98)	<0.001	3.45 (3.28, 3.62)	1.84 (1.69, 2.00)	<0.001
	Referred from informal setting ^	15.44 (11.2, 21.31)	5.06 (3.35, 7.63)	<0.001	4.45 (3.78, 5.25)	2.25 (2.00, 3.10)	<0.001
Cervix dilatation at admission **							
	Latent phase (0–3 cm)	6.66 (5.65, 7.86)	2.32 (1.89, 2.85)	<0.001	1.61 (1.52, 1.70)	1.56 (1.45, 1.67)	<0.001
	*Active phase (reference) (4 or above)*	1	1		1	1	
Birth mode							
	*Spontaneous vaginal birth (reference)*	1	1		1	1	
	Assisted vaginal birth	4.53 (2.58, 7.96)	2.28 (1.24, 4.19)	0.008	3.34 (2.65, 4.20)	1.78 (1.36, 2.31)	<0.001
	Elective caesarean section	0.57 (0.39, 0.82)	0.42 (0.26, 0.70)	0.001	0.35 (0.31, 0.40)	0.28 (0.23, 0.33)	<0.001
	Emergency caesarean section	2.01 (8.61, 11.65)	0.79 (0.61, 1.01)	0.063	1.49 (1.40, 1.58)	0.66 (0.59, 0.73)	<0.001
Labour monitoring tool used ***							
	*Yes (reference)*	1	1		1	1	
	No	7.47 (6.00, 9.29)	4.45 (3.46, 5.73)	<0.001	2.82 (2.63, 3.02)	2.37 (2.17, 2.60)	<0.001
	Unknown	19.95 (15.27, 26.04)	3.92 (2.81, 5.47)	<0.001	3.40 (2.99, 3.87)	1.70 (1.45, 1.99)	<0.001
Companionship in labour							
	*Woman had companion (reference)*	1	1		1	1	
	Woman did not have companion	1.61 (1.25, 2.08)	1.43 (1.07, 1.94)	0.017	1.68 (1.56, 1.81)	1.67 (1.49, 1.87)	<0.001
	It was unknown if the woman had a companion	3.32 (2.56, 4.28)	1.68 (1.23, 2.29)	0.001	1.73 (1.59, 1.89)	1.45 (1.29, 1.64)	<0.001
Woman received uterotonic for PPH prevention **							
	Yes (reference)	1	1		–	–	
	No	2.34 (1.83, 3.01)	2.01 (1.52, 2.68)	<0.001	–	–	
	Unknown	5.43 (4.49, 6.54)	2.26 (1.77, 2.89)	<0.001	–	–	
Facility +							

^±^Outcome of gynaecological or obstetric admission was a maternal death ^§^ Outcome of obstetric admission was a stillbirth or neonatal death. ^∞^ 832 obstetric admissions had unknown data on vital status of baby(ies) at birth and were excluded from the perinatal death analysis model. ª Separate multilevel logistic regressions were used for maternal death and perinatal death. ^ traditional birth attendant, mission home or other informal setting; ** for obstetric admissions only; *** for women without pre-labour caesarean section; + adjusted model accounted for random effects at the facility level; 0.271 and 0.133 was the variation between facilities in maternal and perinatal death (respectively). The model quantified the between and with facility (between women) variations and indicate that there is a substantial variation between facilities over and above the between women variations (*p*<0.001).

The independent predictors of maternal death were: low maternal education, no antenatal care or antenatal care received outside of a health facility, referral from other facility or informal setting, previous caesarean section, admission during the latent phase of labour, operative vaginal birth, non-use of labour monitoring tool, no companion present during labour, and non-use of uterotonic for postpartum haemorrhage (PPH) prevention.

Predictors of perinatal death were: maternal age >35 year of age, low maternal education, no antenatal care or antenatal care outside of a health facility, referral from other facility or informal setting, previous caesarean section, admission during the latent phase of labour, operative vaginal birth, non-use of labour monitoring tool, no companion present during labour, and non-use of uterotonic for PPH prevention.

### Missing data and sensitivity analyses

There were few missing data. We do not have any specific information on the three publicly-funded facilities that did not participate in the study, therefore we were unable to ascertain if the missing data would be missing at random. All individual variables had <5% missing data, except for women's education level (7·8%). Women's education was found to be missing at random. We conducted a sensitivity analysis using only complete cases for women's education level and compared estimates to the multilevel model that included all cases. There was very little impact on the magnitude or direction in odds ratios and statistical significance. Although all variables in the model had less than <5% missing data, three important variables, use of labour monitoring tool, companionship in labour and uterotonic for PPH prevention, had responses that could fall in an ‘unknown’ category. We ran a sensitivity analysis with all women/babies with unknown data on use of labour monitoring tool, companionship in labour or uterotonic for PPH prevention removed from the maternal and perinatal death models (*n* = 17,836 cases with unknown data were removed), and found that these interventions were still associated with death with similar effect estimates. We also ran a sensitivity analysis with women/babies from privately-funded facilities removed from the model. The effect estimates did not change substantially.

## Discussion

Our study reveals a high burden of severe maternal and perinatal complications in Nigerian referral-level facilities. Close to one-tenth of the women admitted for delivery or on account of pregnancy-related complications had a potentially life-threatening event and an unacceptably high proportion of these women died. Maternal and perinatal mortality were particularly high amongst women who were referred, and those who had already developed potentially life-threatening complications prior to hospital admission. Delays in reaching the hospital and delays in receiving care upon arrival at the hospital were the most commonly attributed avoidable factors contributing to maternal and perinatal deaths. The lack of antenatal care at the same facility, referral, lack of a labour companion, non-use of labour monitoring tool, and non-use of uterotonic for PPH prophylaxis, independently increased the odds of maternal and perinatal deaths. Our quality of care indicators revealed suboptimal coverage of labour companionship and use of uterotonic for PPH prevention. In addition, while eclampsia and PPH were the most frequent complications observed during the study period, the survival rates following maternal sepsis and eclampsia were considerably lower than for other complications.

To our knowledge, this study reports the largest dataset of individual-level data on women and newborns admitted to referral-level facilities in Nigeria and the region. It was the first attempt to investigate several previously unexplored interventions as potential predictors for maternal and perinatal death, which was possible because of the large data set and inclusion of information for women with complicated and uncomplicated pregnancies.

A number of limitations need to be acknowledged. Despite the use of a manual of operating procedures to standardise implementation of the study across hospitals, the large number of hospitals, medical staff, clinical protocols, and patient medical record formats in use across the network may have resulted in misclassification or heterogeneity in documentation of pregnancy-related events, and the incompleteness of important data. Although our best efforts were made to include a range of predictors that may influence maternal and perinatal death residual confounding may be present. For example, pregnancy weight gain, use of traditional herbal medicines during pregnancy, alcohol and drug use were not captured in our data, so could not be explored. Finally, our study was conducted in a hospital network that largely represents publicly-funded referral-level tertiary facilities in Nigeria, and despite the inclusion of consenting private health facilities, our data might not reflect the quality of care in the private health sector, or at lower level health facilities.

The disproportionately high burden of maternal and perinatal mortality reported in this study suggests that facility-based births in this setting are still associated with suboptimal quality of care. Since the conduct of the Nigerian Near-miss and Maternal Death Survey more than five years ago,^5^ it appears that there has been little or no improvement in maternal mortality reduction at this level of health care delivery in Nigeria. The high intra-hospital maternal mortality ratio (1088 per 100,000 live births, 1·1%) reported in the near-miss study (comprised of publicly-funded facilities) is comparable to 1.3% seen in the current study for publicly-funded facilities. In the global context, this burden of maternal mortality in Nigerian referral-level hospitals, either in relative or absolute terms, surpassed those observed from similar surveillance systems in other low- and middle-income countries.^13^^,^^14^

The contributory factors to maternal and perinatal mortality were largely similar to previous observations in the literature.^15^, ^16^, ^17^, ^18^, ^19^ About half of women and babies who died in our study occurred in situations where the woman was referred for care at the participating hospitals, with one in five arriving with a potentially life-threatening complication or having deteriorated to a state where the condition was life-threatening. Studies in LMICs show that delay in transport is a contributing factor in around one-third of maternal deaths.^20^^,^^21^ Previous studies have highlighted poor and inefficient referral systems in Nigeria and other LMICs.^18^^,^^19^^,^^22^^,^^23^ The delays in referral or delay in transport to facility, combined with suboptimal emergency readiness to deal with life-threatening complications at these hospitals were likely responsible for the overall poor maternal and perinatal outcomes. The difference in mortality rates between privately-funded and publicly-funded facilities is likely due to differences in sociodemographic characteristics.

The most striking findings in our study were the strong associations between maternal and perinatal deaths and the lack of a labour companion, non-use of labour monitoring tool, and non-use of uterotonic for PPH prevention. While systematic reviews of randomised trials on labour companionship, partograph, and uterotonics for PPH prevention (compared with no intervention or usual care) have reported findings that were consistent with general improvement in important maternal and/or perinatal outcomes, none have reported impact on mortality outcomes.^24^, ^25^, ^26^ This may be due to the prohibitively large sample sizes and high event rates that would be needed to detect such important differences between comparison groups. More research is needed to confirm this finding in other settings.

We found high case fatality rates for sepsis and eclampsia, suggesting that on average, about one of every four women with eclampsia and four of every ten women with sepsis did not survive their underlying complications. These findings are consistent with the observations in Nigeria Near-miss and Maternal Death Survey where maternal systemic infections and eclampsia also had the worst outcomes.^5^ The underlying reasons for these persistent findings are likely to be multifactorial though would be most likely related to delays in presentation to the hospital and delays in treatment upon arrival at the hospital. Such contributory factors were reported previously in the Nigeria Near-miss and Maternal Death Survey.^5^ Not only do sepsis and eclampsia require appropriate interventions (antibiotics and magnesium sulphate) to avert a maternal death, but also a timely administration of such interventions before end-stage organ damage ensues. This assertion is supported by the general availability of magnesium sulphate in the majority of the participating hospitals, which did not translate to improved outcomes for women with eclampsia. The situation is further compounded by the lack of capacity to initiate magnesium sulphate administration at lower level facilities (or sources of referral) due to complex dosing regimens, fear of magnesium toxicity, or regulatory issues, such that women referred with severe pre-eclampsia or eclampsia to referral-level facilities arrive when magnesium sulphate can make little or no difference to maternal survival.^27^^,^^28^

Our study provided the opportunity to compare a range of quality of care indicators between hospitals and regions within Nigeria, and across WHO QED countries. The reported QED indicators provided important insight into the quality of care provided to women and babies who received care at the participating hospitals during the study period. Notably, two of the QED indicators, proportions of women who had a labour companion and who received uterotonic for prevention of postpartum haemorrhage were strongly associated with both maternal and perinatal death, indicating where short-term effort could lead to substantial mortality reduction. Use of these QED indicators should continue. We found that “pre-discharge” maternal mortality ratio was not an optimal indicator for assessment of the overall standard of care provided at the participating hospitals, as it did not consider maternal deaths amongst women who already gave birth prior to admission to these hospitals. The huge disparity between intrahospital maternal mortality ratio (1361 per 100,000 live births) and the rate of pre-discharge maternal death (683 per 100,000 live births), albeit using different denominators, highlights the importance of including all women managed in a hospital in the indicators of facility performance, particularly in settings where referrals of critically-ill women are high.

To ensure survival of women and neonates amongst those presenting with life-threatening complications, the quality of care at Nigerian referral-level hospitals needs to improve, particularly with regard to the capacity to manage critically ill referrals. However, real change in maternal and perinatal health profile will be dependant on the country's ability to improve emergency obstetric and neonatal care at lower-level facilities and strengthening of the referral network to reduce the burden of potentially life-threatening complications that overwhelms the tertiary health care system. The observed proportion of maternal death due to eclampsia calls for research to identify simpler magnesium sulfate regimens which can be promptly initiated at lower-level facilities.

As the predictors of death were similar for women and neonates, quality of care improvement strategies should be focused on the mother-infant dyad in a way that allows for an integrated approach for optimised labour, childbirth and early neonatal care. For example, a targeted approach in increasing companionship in labour will not only increase the practice of respectful care, but also improve maternal and perinatal survival. The low rate of companionship during labour in this study, coupled with the strong association with maternal survival, presents an opportunity for instituting impactful quality-of-care improvement strategies.^29^ In Nigeria, it is not usual practice for spouses to accompany women while in labour.^30^ Companionship in labour may be facilitated by providing an enabling environment both structurally and socially, including privacy and curtains for each birthing woman, and ensuring that hospital policies and procedures support labour companionship. On the longer-term, a multi-sectoral collaboration (for example, to increase education for women and girls, increase awareness on the importance of timely care for obstetric complications through local media campaigns, and improve road and transport infrastructure) is needed to ensure all women and their babies receive quality care to not only survive but thrive and achieve their full life potential. This will require a collaborative effort across Ministry of Education, Ministry of Health, Ministry of Labour and Employment, Ministry of Information and Culture and Ministry of Transportation.

Prospective surveillance of maternal and perinatal data, and use of selected indicators for periodic assessment of hospital performance and quality improvement is critical for achieving the aims of the WHO QED initiative at the country level. In the future, electronic databases could strengthen the understanding of referral pathways and maternal death audit review by capturing and linking information on women at the primary, secondary and tertiary level. A nationwide database programme for harmonizing and aggregating data could be implemented in settings with similar medical record infrastructure as Nigeria, to determine easily achievable interventions that can drive policy change and impact. With the global shift towards increased facility births and the digitization of routine national health management systems, there is a huge potential to scale up this programme to other settings. Such investment in prospective surveillance of maternal and perinatal data and quality improvement is critical for achieving the aims of the third sustainable development goal targets.

## Funding

This work was funded by MSD for Mothers; and UNDP/UNFPA/ UNICEF/WHO/World Bank Special Programme of Research, Development and Research Training in Human Reproduction (HRP), a co-sponsored programme executed by the World Health Organization (WHO).

## Author contributions

The implementation of this project was a collaborative effort of a large number of academic staff, hospital personnel and researchers from 54 referral-level Nigeria hospitals – The Maternal and Perinatal Database for Quality, Equity and Dignity (MPD-4-QED) Network. OTO conceived the study. TL and JT drafted the protocol for the analysis of the database with substantial input from OTO. TL and MB prepared the statistical analysis plan and led all statistical analysis. All authors reviewed and interpreted the data at a workshop convened by WHO. JT, TL, and OTO led and coordinated the writing of the manuscript. The writing committee (AA, MA, KB, ME, AG, HI, OI, SK, LNO, EN, TOO, LO, KT, MB, IA, BE, PA, BA, CC, SE, HG, JI) wrote and provided feedback on the first draft. JT, TL, and OTO revised and consolidated the final manuscript. All authors and named members of MPD-4-QED Network had an opportunity to revise the manuscript for intellectual content and approved it for publication. The manuscript represents the views of the named authors only and does not reflect the views of MSD for Mothers, the UNDP/UNFPA/UNICEF/WHO/World Bank Special Programme of Research, Development and Research Training in Human Reproduction (HRP) or the World Health Organization.

## Data sharing statement

All relevant data are within the manuscript and its Supporting Information files.

## Declaration of interests

The authors declare no competing interests.

## Appendix Supplementary materials

Image, application 1

Image, application 2
