# Supplementary material for: Quality and outcomes of maternal and perinatal care for 76,563 pregnancies reported in a nationwide network of Nigerian referral-level hospitals
Source: eClinicalMedicine. 2022 Apr 28;47:101411. doi: 10.1016/j.eclinm.2022.101411 (PMC9065588; doi:10.1016/j.eclinm.2022.101411)
Supplement: Supplementary file 1 [file mmc1.docx]

**Supplementary Tables and Figures**

**Table S1. Summary of the key features of the *Maternal and Perinatal Database for Quality, Equity and Dignity* Programme**

| *Data collection*   - Both maternal and perinatal data collected (prevents duplication of data collection systems) - Customized existing open-source District Health Information Software (DHIS-2) - Tablet-based electronic system - Data entered by medical record officers (not nurses, midwives or doctors) - Data collection easily implemented into existing data collection processes - No contact with mothers, so consent not needed - Information collected can be adapted (COVID-19 items added at beginning of pandemic)   *Data analysis*   - Assessment of burden of complications at the facility, regional and national level - Causes of death for fetal losses <28 weeks, stillbirth, neonatal death and maternal death in the one system - Predictors of death could be examined due to inclusion of healthy pregnancies - ICD-PM could be easily applied as data linked as between mother and neonate - Few missing data (e.g. for vital status <0.1%)   *Data use for quality improvement*   - Direct comparison of quality of care indicators between hospitals and regions - Multiple uses of the data including interface for facilities for quality improvement and ability to export data to statistical analysis programs - Quality of care indicators can be reviewed across the spectrum of healthy and complicated pregnancies - Additional quality of care indicators can be added to align with global and national initiatives |
| --- |

**Table S2. Available human resources by hospital admissions, live births and maternal and perinatal mortality outcomes**

|  | **Strength of workforce (n)** | | | | | | | | | **Number of admissions, births and deaths*** | | | | | |
| --- | --- | --- | --- | --- | --- | --- | --- | --- | --- | --- | --- | --- | --- | --- | --- |
| **Location of facility** | **Obstetric consultants** | **Obstetric resident doctors** | **Obstetric house officers** | **Labour ward nurses** | **Obstetric nurses** | **Neonatal consultants** | **Neonatal resident doctors** | **Neonatal house officers** | **Neonatal nurses** | **Women admitted ∞** | **Maternal deaths** | **Live births *** | **Stillbirths**** | **Neonatal deaths ***** | ***Missing vital status at birth*** |
| **Northcentral** | 9 | 28 | 9 | 26 | 16 | 2 | 3 | 3 | 21 | 2439 | 15 | 2237 | 75 | 14 | *5* |
|  | 9 | 19 | 10 | 18 | 6 | 2 | 5 | 2 | 16 | 1182 | 20 | 1000 | 48 | 18 | *14* |
|  | 15 | 28 | 17 | 18 | 6 | 5 | 9 | 6 | 28 | 1807 | 27 | 1616 | 124 | 35 | *26* |
|  | 14 | 44 | 15 | 35 | 0 | 2 | 7 | 4 | 23 | 877 | 18 | 716 | 58 | 12 | *4* |
|  | 7 | 22 | 4 | 28 | 0 | 1 | 5 | 2 | 10 | 2506 | 26 | 2129 | 207 | 4 | *52* |
|  | 5 | 23 | 5 | 15 | 3 | 4 | 4 | 5 | 15 | 1190 | 22 | 1078 | 107 | 5 | *9* |
|  | 18 | 42 | 18 | 14 | 9 | 4 | 6 | 3 | 15 | 1341 | 17 | 1236 | 48 | 8 | *20* |
|  | 18 | 33 | 20 | 17 | 12 | 4 | 6 | 6 | 24 | 1758 | 21 | 1566 | 95 | 48 | *22* |
|  | 12 | 34 | 26 | 17 | 9 | 4 | 13 | 6 | 24 | 915 | 10 | 859 | 50 | 35 | *3* |
| **Regional total** | **107** | **273** | **124** | **188** | **61** | **28** | **58** | **37** | **176** | **14015** | **176** | **12437** | **812** | **179** | ***155*** |
| **Northwest** | 4 | 1 | 1 | 3 | 3 | 2 | 1 | 1 | 2 | 872 | 34 | 601 | 88 | 22 | *79* |
|  | 26 | 56 | 18 | 17 | 0 | 4 | 6 | 2 | 18 | 684 | 20 | 605 | 86 | 4 | *11* |
|  | 3 | 7 | 12 | 23 | 5 | 2 | 2 | 2 | 13 | 2098 | 23 | 1937 | 118 | 9 | *7* |
|  | 19 | 34 | 8 | 20 | 11 | 3 | 4 | 2 | 20 | 3118 | 28 | 2832 | 193 | 59 | *50* |
|  | 4 | 4 | 0 | 8 | 2 | 1 | 2 | 2 | 4 | 336 | 2 | 317 | 8 | 0 | *0* |
|  | 5 | 6 | 0 | 13 | 1 | 2 | 0 | 0 | 2 | 655 | 1 | 622 | 7 | 4 | *1* |
|  | *-* | - | - | - | - | - | - | - | - | 2184 | 14 | 2073 | 104 | 15 | *12* |
|  | 5 | 18 | 4 | 22 | 6 | 3 | 13 | 4 | 21 | 1689 | 56 | 1347 | 183 | 48 | *38* |
|  | 14 | 29 | 1 | 18 |  | 3 | 5 | 3 | 21 | 3137 | 43 | 2802 | 105 | 16 | *19* |
|  | 7 | 20 | 4 | 16 | 7 | 2 | 3 | 1 | 17 | 2210 | 33 | 1945 | 179 | 53 | *27* |
| **Regional total** | **87** | **175** | **48** | **140** | **35** | **22** | **36** | **17** | **118** | **16983** | **254** | **15081** | **1071** | **230** | ***244*** |
| **Northeast** | 4 | 12 | 4 | 10 | 6 | 2 | 2 | 1 | 6 | 1425 | 33 | 1324 | 121 | 25 | *21* |
|  | 4 | 25 | 9 | 13 | 10 | 4 | 2 | 2 | 8 | 3112 | 54 | 2628 | 427 | 66 | *63* |
|  | 4 | 14 | 2 | 15 | 4 | 4 | 2 | 2 | 4 | 1500 | 37 | 1091 | 249 | 48 | *71* |
|  | 10 | 12 | 4 | 12 | 6 | 4 | 6 | 2 | 6 | 2533 | 24 | 2465 | 78 | 32 | *4* |
|  | 9 | 19 | 12 | 12 | 3 | 2 | 12 | 9 | 19 | 2156 | 14 | 2029 | 112 | 21 | *0* |
|  | 5 | 12 | 4 | 20 | 6 | 2 | 2 | 1 | 4 | 1402 | 17 | 1229 | 45 | 27 | *7* |
|  | 3 | 18 | 12 | 18 | 10 | 2 | 12 | 4 | 8 | 1294 | 41 | 948 | 165 | 26 | *52* |
| **Regional total** | **39** | **112** | **47** | **100** | **45** | **20** | **38** | **21** | **55** | **13422** | **220** | **11714** | **1197** | **245** | ***218*** |
| **Southwest** | 7 | 13 | 8 | 15 | 18 | 1 | 2 | 2 | 15 | 1356 | 5 | 1277 | 57 | 18 | *7* |
|  | 15 | 54 | 16 | 22 | 12 | 3 | 10 | 16 |  | 714 | 17 | 643 | 57 | 29 | *11* |
|  | 22 | 36 | 18 | 18 | 9 | 2 | 3 | 2 | 17 | 620 | 4 | 569 | 29 | 4 | *0* |
|  | 10 | 27 | 20 | 15 | 15 | 3 | 5 | 6 | 21 | 1152 | 10 | 1095 | 35 | 22 | *6* |
|  | 10 | 19 | 8 | 8 | 5 | 2 | 3 | 3 | 8 | 441 | 7 | 414 | 19 | 4 | *2* |
|  |  |  |  |  |  |  |  |  |  | 2643 | 19 | 2516 | 125 | 55 | *5* |
|  | 5 | 20 | 16 | 13 | 12 | 2 | 11 | 16 | 15 | 689 | 4 | 615 | 35 | 2 | *0* |
|  | 16 | 27 | 12 | 19 | - | 2 | 2 | 3 | 20 | 2169 | 23 | 2019 | 100 | 23 | *31* |
|  | 21 | 36 | 16 | 58 | - | 2 | 4 | 3 | 34 | 1266 | 22 | 1212 | 37 | 17 | *1* |
|  | 1 | 1 | 0 | 6 | 2 | 1 | 1 | 0 | - | 424 | 0 | 388 | 5 | 3 | *0* |
|  | *-* | - | - | - | - | - | - | - | - | 646 | 4 | 597 | 19 | 3 | *1* |
|  | *-* | - | - | - | - | - | - | - | - | 2461 | 1 | 2312 | 73 | 6 | *2* |
| **Regional total** | **107** | **233** | **114** | **174** | **73** | **18** | **41** | **51** | **130** | **14581** | **116** | **13657** | **591** | **186** | ***66*** |
| **Southsouth** | 18 | 31 | 14 | 16 | 3 | 2 | 4 | 6 | 23 | 1002 | 11 | 928 | 66 | 11 | *6* |
|  | 24 | 40 | 7 | 20 | 11 | 4 | 12 | 4 | 32 | 1759 | 23 | 1527 | 94 | 20 | *26* |
|  | 26 | 60 | 6 | 28 | 5 | 3 | 7 | 2 | 22 | 1261 | 28 | 1064 | 83 | 24 | *23* |
|  | 10 | 17 | 6 | 24 | 8 | 2 | 4 | 2 | 10 | 174 | 9 | 134 | 14 | 5 | *7* |
|  | 18 | 29 | 33 | 28 | 13 | 3 | 7 | 11 | 32 | 1841 | 40 | 1707 | 99 | 45 | *19* |
|  | 16 | 27 | 9 | 21 | 16 | 2 | 5 | 4 | 21 | 890 | 3 | 850 | 36 | 23 | *5* |
|  | 1 | 7 | 0 | 14 | 12 | 1 | 8 | 0 | 10 | 1243 | 1 | 1220 | 26 | 12 | *0* |
|  | 2 | 4 | 0 | 14 | 5 | 2 | 4 | 0 | 7 | 775 | 1 | 713 | 5 | 3 | *3* |
|  | 9 | 7 | 9 | 9 | 9 | 4 | 2 | 3 | 10 | 362 | 4 | 304 | 19 | 2 | *2* |
| **Regional total** | **124** | **222** | **84** | **174** | **82** | **23** | **53** | **32** | **167** | **9307** | **120** | **8447** | **442** | **145** | ***91*** |
| **Southeast** | 14 | 26 | 11 | 14 | 14 | 2 | 6 | 3 | 14 | 1255 | 7 | 1151 | 59 | 5 | *1* |
|  | 20 | 37 | 29 | 18 | 18 | 5 | 6 | 10 | 18 | 744 | 8 | 656 | 41 | 17 | *15* |
|  | *-* | - | - | - | - | - | - | - | - | 843 | 6 | 776 | 28 | 12 | *9* |
|  | *-* | - | - | - | - | - | - | - | - | 1113 | 13 | 996 | 47 | 5 | *12* |
|  | 36 | 46 | 17 | 16 | 4 | 4 | 7 | 2 | 18 | 392 | 4 | 357 | 24 | 15 | *2* |
|  | *-* | - | - | - | - | - | - | - | - | 1556 | 6 | 1513 | 86 | 9 | *0* |
|  | *-* | - | - | - | - | - | - | - | - | 2352 | 10 | 2270 | 100 | 42 | *19* |
| **Regional total** | **70** | **109** | **57** | **48** | **36** | **11** | **19** | **15** | **50** | **8255** | **54** | **7719** | **385** | **105** | ***58*** |
| **MPD-4-QED Network total** | **534** | **1124** | **474** | **824** | **332** | **122** | **245** | **173** | **696** | **76563** | **940** | **69055** | **4498** | **1090** | ***832*** |

∞ Number of women admitted to facility for delivery or on account of complications within 42 days of delivery or termination of pregnancy * number of live births to women who were admitted to facility (includes babies born inside and outside of facility); 67,971 live births occurred in facility ** number of stillbirths to women who were admitted to facility, 4284 stillbirths occurred in facility ***number of neonatal deaths to women who were admitted (includes babies born in and outside of hospital), 1,043 babies were born in facility and died before discharge

**Table S3. Cause specific case fatality rate (CFR) for main causes of death across the network and by region**

|  | **All hospitals** | | | **Northcentral** | | | **Northeast** | | | **Northwest** | | | **Southeast** | | | **Southsouth** | | | **Southwest** | | |
| --- | --- | --- | --- | --- | --- | --- | --- | --- | --- | --- | --- | --- | --- | --- | --- | --- | --- | --- | --- | --- | --- |
|  | **Women with complication, deaths (n)** | | **CFR** | **Women with complication, deaths (n)** | | **CFR** | **Women with complication, deaths (n)** | | **CFR** | **Women with complication, deaths (n)** | | **CFR** | **Women with complication, deaths (n)** | | **CFR** | **Women with complication, deaths (n)** | | **CFR** | **Women with complication, deaths (n)** | | **CFR** |
| **Obstetric haemorrhage** |  |  |  |  |  |  |  |  |  |  |  |  |  |  |  |  |  |  |  |  |  |
| Placenta praevia | 556 | 14 | 2.5 | 154 | 4 | 2.6 | 106 | 2 | 1.9 | 80 | 2 | 2.5 | 31 | 2 | 6.5 | 73 | 1 | 1.4 | 112 | 3 | 2.7 |
| Abruptio placentae | 919 | 33 | 3.6 | 202 | 5 | 2.5 | 345 | 11 | 3.2 | 171 | 12 | 7.0 | 32 | 1 | 3.1 | 71 | 1 | 1.4 | 98 | 2 | 2.0 |
| Postpartum haemorrhage (EBL >500mL) | 9884 | 103 | 1.0 | 2408 | 23 | 1.0 | 904 | 20 | 2.2 | 1605 | 27 | 1.7 | 987 | 2 | 0.2 | 1906 | 7 | 0.4 | 2074 | 24 | 1.2 |
| **Pregnancy-related infection** |  |  |  |  |  |  |  |  |  |  |  |  |  |  |  |  |  |  |  |  |  |
| Puerperal sepsis | 240 | 98 | 40.8 | 41 | 19 | 46.3 | 74 | 26 | 35.1 | 40 | 18 | 45.0 | 8 | 4 | 50.0 | 46 | 20 | 43.5 | 31 | 11 | 35.5 |
| **Abortive outcome** |  |  |  |  |  |  |  |  |  |  |  |  |  |  |  |  |  |  |  |  |  |
| Haemorrhage in early pregnancy | 254 | 4 | 1.6 | 17 | 1 | 5.9 | 78 | 2 | 2.6 | 66 | 1 | 1.5 | 35 | 0 | 0.0 | 36 | 0 | 0.0 | 22 | 0 | 0.0 |
| Ectopic pregnancy | 722 | 7 | 1.0 | 155 | 2 | 1.3 | 73 | 0 | 0.0 | 129 | 1 | 0.8 | 50 | 1 | 2.0 | 128 | 1 | 0.8 | 187 | 2 | 1.1 |
| **Hypertensive disorders** |  |  |  |  |  |  |  |  |  |  |  |  |  |  |  |  |  |  |  |  |  |
| Any hypertensive disorder | 5157 | 289 | 5.6 | 706 | 46 | 4.3 | 735 | 75 | 7.3 | 689 | 74 | 8.1 | 175 | 13 | 4.3 | 468 | 33 | 5.0 | 666 | 17 | 2.1 |
| Pre-existing hypertension | 1718 | 24 | 1.4 | 435 | 3 | 0.7 | 360 | 5 | 1.4 | 313 | 7 | 2.2 | 152 | 1 | 0.7 | 214 | 1 | 0.5 | 244 | 2 | 0.8 |
| Pregnancy-induced hypertension | 2689 | 70 | 2.6 | 583 | 16 | 2.7 | 476 | 28 | 5.9 | 513 | 8 | 1.6 | 144 | 7 | 4.9 | 387 | 8 | 2.1 | 586 | 4 | 0.7 |
| Eclampsia | 750 | 187 | 24.9 | 123 | 30 | 24.4 | 259 | 47 | 18.1 | 176 | 66 | 37.5 | 31 | 6 | 19.4 | 81 | 25 | 30.9 | 80 | 13 | 16.3 |
| **Obstructed labour** |  |  |  |  |  |  |  |  |  |  |  |  |  |  |  |  |  |  |  |  |  |
| Obstructed labour due to any cause | 1200 | 22 | 1.8 | 201 | 3 | 1.5 | 302 | 2 | 0.7 | 225 | 4 | 1.8 | 95 | 2 | 2.1 | 224 | 8 | 3.6 | 153 | 3 | 2.0 |

**Table S4. Causes of perinatal deaths for babies born in a *Maternal and Perinatal Database for Quality, Equity and Dignity* Programme hospital using the WHO application of ICD-10 to deaths during the perinatal period: ICD-perinatal mortality (ICD-PM)(n=4,667 audited perinatal deaths)**

| **Perinatal condition +** | **M1: Complications of placenta, cord and membranes** | **M2: Maternal complications of pregnancy** | **M3: Other complications of labour and delivery** | **M4: Maternal and medical surgical conditions** | **M5: No maternal condition** | **Total (%)** |
| --- | --- | --- | --- | --- | --- | --- |
| **Antepartum death** |  |  |  |  |  |  |
| A1: Congenital malformations, deformations and chromosomal abnormalities | 8 | 17 | 3 | 11 | 85 | 124 (4.7) |
| A2: Infection | 32 | 46 | 7 | 23 | 12 | 120 (4.6) |
| A3: Antepartum hypoxia | 441 | 573 | 118 | 152 | 171 | 1455 (55.5) |
| A4: Other specified antepartum disorder | 65 | 150 | 19 | 36 | 27 | 297 (11.3) |
| A5: Disorders related to fetal growth | 10 | 29 | 5 | 13 | 19 | 76 (2.9) |
| A6: Fetal death of unspecified cause | 28 | 91 | 26 | 55 | 351 | 551 (21.0) |
| **Total** | **584** | **906** | **178** | **290** | **665** | **2623 (100)** |
| **Intrapartum death** |  |  |  |  |  |  |
| I1: Congenital malformations, deformations and chromosomal abnormalities | 2 | 4 | 1 | 1 | 27 | 35 (3.1) |
| 12: Birth trauma | 0 | 0 | 8 | 0 | 3 | 11 (1.0) |
| 13: Acute intrapartum event | 272 | 125 | 319 | 48 | 149 | 913 (79.8) |
| I4: Infection | 6 | 2 | 2 | 1 | 5 | 16 (1.4) |
| I5: Other specified intrapartum disorder | 3 | 7 | 32 | 6 | 9 | 57 (4.9) |
| I6: Disorders related to fetal growth | 1 | 8 | 1 | 3 | 12 | 25 (2.2) |
| I7: Intrapartum death of unspecified cause | 8 | 14 | 20 | 5 | 40 | 87 (7.6) |
| **Total (%)** | **292** | **160** | **383** | **64** | **245** | **1144 (100)** |
| **Neonatal death** |  |  |  |  |  |  |
| N1: Congenital malformations, deformations and chromosomal abnormalities | 1 | 4 | 2 | 4 | 27 | 38 (4.2) |
| N2: Disorders related to fetal growth | 0 | 9 | 3 | 3 | 2 | 17 (1.9) |
| N3: Birth trauma | 0 | 1 | 1 | 0 | 3 | 5 (0.6) |
| N4: Complications of intrapartum events | 45 | 41 | 238 | 26 | 66 | 416 (46.2) |
| N5: Convulsions and disorders of cerebral status | 2 | 2 | 3 | 2 | 3 | 12 (1.3) |
| N6: Infection | 4 | 1 | 1 | 1 | 4 | 11 (1.2) |
| N7: Respiratory and cardiovascular disorders | 4 | 10 | 6 | 7 | 13 | 40 (4.4) |
| N8: Other neonatal conditions | 0 | 1 | 3 | 1 | 8 | 13 (1.4) |
| N9: Low birthweight and prematurity | 64 | 123 | 11 | 38 | 76 | 312 (34.7) |
| N10: Miscellaneous | 2 | 3 | 0 | 3 | 3 | 11 (1.2) |
| N11: Neonatal death of unspecified cause | 2 | 7 | 0 | 3 | 13 | 25 (2.9) |
| **Total (%)** | **124** | **202** | **268** | **88** | **218** | **900 (100)** |

+ Percentages are within antepartum, intrapartum and neonatal categories
