# Supplementary material for: Quality and outcomes of maternal and perinatal care for 76,563 pregnancies reported in a nationwide network of Nigerian referral-level hospitals
Source: eClinicalMedicine. 2022 Apr 28;47:101411. doi: 10.1016/j.eclinm.2022.101411 (PMC9065588; doi:10.1016/j.eclinm.2022.101411)
Supplement: Supplementary file 2 [file mmc2.docx]

Appendices.

**APPENDIX I. Names and affiliation of MPD-4-QED Collaborators**

| **Name** | **Affiliation** |
| --- | --- |
| Abiodun S Adeniran | University of Ilorin Teaching Hospital, Ilorin, Nigeria. |
| Aishatu A Gobir | University of Ilorin Teaching Hospital, Ilorin,  Nigeria |
| Amaka Ocheke | Jos University Teaching Hospital, Jos, Nigeria |
| Fatimah Baba Joy | Jos University Teaching Hospital, Jos, Nigeria |
| Ibrahim Rais | National Hospital, Federal Capital Territory, Abuja, Nigeria |
| Amsa B Mairami | National Hospital, Federal Capital Territory, Abuja, Nigeria |
| Mohammed S. Ozegya | Dalhatu Araf Specialist Hospital, Lafia, Nigeria |
| Samuel Pam | Federal Medical Centre, Keffi, Nigeria |
| Sarah Ango | Federal Medical Centre, Keffi, Nigeria |
| Musa Abdulkarim Omoyine | Federal Medical Centre, Lokoja, Nigeria |
| Medupin Patricia | Federal Medical Centre, Lokoja, Kogi Stage, Nigeria |
| Silas Ochejele | Federal Medical Center, Makurdi, Nigeria. |
| Egwu Agada | Federal Medical Center, Makurdi, Nigeria. |
| Duum Nwachukwu | Federal Medical Centre, Bida, Nigeria |
| Grace Ahmed | Federal Medical Centre, Bida, Nigeria |
| Aisha Abdurrahman | Federal Medical Centre, Katsina, Nigeria |
| Lawal M Ibrahim | Federal Medical Centre, Katsina, Nigeria |
| Aisha Nana Adamu | Federal Medical Centre, Birnin Kebbi, Nigeria |
| Aliyu Na'uzo | Federal Medical Centre, Birnin Kebbi, Nigeria |
| Adewale Ashimi | Federal Medical Centre, Birnin Kudu, Nigeria |
| Umma Idris | Federal Medical Centre, Birnin Kudu, Nigeria |
| Owodunni A Adebola | Federal Medical Centre, Gusau, Nigeria |
| Festus D Akeredolu | Federal Medical Centre, Gusau, Nigeria |
| Asma’u Adamu | Uthman Danfodiyo University Teaching Hospital,  Sokoto, Nigeria |
| Aliyu Labaran | Aminu Kano Teaching Hospital, Kano, Nigeria |
| Adekunle Oguntayo | Ahmadu Bello University Teaching Hospital, Zaria, Nigeria |
| Abdulkadir Isa | Ahmadu Bello University Teaching Hospital, Zaria, Nigeria |
| Stephen Bature | Kaduna State University Teaching Hospital, Kaduna, Nigeria |
| Andeyantso E Ayuba | Ahmadu Bello University Teaching Hospital, Zaria, Nigeria |
| Hauwa Abdullahi | Getwell Hospital, Kano, Nigeria |
| Zubaida L Farouk | Getwell Hospital, Kano, Nigeria |
| Sulaiman Muhammad Daneji | Standard Specialist Hospital, Kano, Nigeria |
| Umar Isa | Standard Specialist Hospital, Kano, Nigeria |
| Samuel Adelaiye | Federal Medical Centre, Azare, Nigeria |
| Ismail M Kalle | Federal Medical Centre, Azare, Nigeria |
| Saidu A Kadas | Abubakar Tafawa Balewa University Teaching Hospital, Bauchi, Nigeria |
| Muhammad F Bashir | Abubakar Tafawa Balewa University Teaching Hospital, Bauchi, Nigeria |
| Joel Moruppa | Federal Medical Center, Yola, Nigeria. |
| Wasinda S Bulus | Federal Medical Center, Yola, Nigeria. |
| Usman R. Yahaya | Federal Medical Center, Gombe, Nigeria |
| Jalo Iliya | Federal Medical Center, Gombe, Nigeria |
| Abdulkarim Mairiga | University of Maiduguri Teaching Hospital, Maiduguri, Nigeria |
| Adamu Atterwahmie | Federal Medical Centre, Nguru, Nigeria |
| Abdulhakeem Hamza | Federal Medical Centre, Nguru, Nigeria |
| Ishaya Wanonyi | Federal Medical Center, Jalingo, Nigeria |
| Uniga A  John | Federal Medical Center, Jalingo, Nigeria |
| Wole Ayegbusi | Obafemi Awolowo University Teaching Hospital Complex, Ile-Ife, Nigeria |
| Adefemi Ayodeji | Lagos State University Teaching Hospital, Ikeja, Nigeria |
| Zainab Imam | Lagos State University Teaching Hospital, Ikeja, Nigeria |
| Opeyemi Akinajo | Lagos University Teaching Hospital, Idi-Araba, Nigeria. |
| Iretiola Fajolu | Lagos University Teaching Hospital, Idi-Araba, Nigeria. |
| Olufemi Akinsanya | Federal Medical Centre, Owo, Nigeria |
| Efeturi Agelebe | Federal Medical Centre, Owo, Nigeria |
| Timothy Oluwasola | University College Hospital Ibadan Nigeria |
| Olukemi O Tongo | University College Hospital Ibadan Nigeria |
| Olusoji Jagun | Olabisi Onabanjo University Teaching Hospital, Sagamu, Nigeria |
| Kuponiyi Opeyemi | Olabisi Onabanjo University Teaching Hospital, Sagamu, Nigeria |
| Olumide Kuku | Molly Specialist Hospital, Ibadan |
| Abimbola Akindolire | Molly Specialist Hospital, Ibadan |
| David O Awonuga | Federal Medical Centre, Abeokuta, Nigeria |
| Iyabode Olabisi F. Dedeke | Federal Medical Centre, Abeokuta, Nigeria |
| Francis Akinkunmi | Mother and Child Hospital Ondo, Nigeria. (now University of Medical Sciences Teaching Hospital, Ondo state, Nigeria |
| Babatunde Olofinbiyi | Ekiti State University Teaching Hospital, Nigeria |
| Ogundare E Olatunde | Ekiti State University Teaching Hospital, Nigeria |
| Olufemi Aworinde | Bowen University, Ogbomoso, Nigeria |
| Efeturi Agelebe | Bowen University, Ogbomoso, Nigeria |
| Olusoji Adeyanju | Adeoyo Maternity Hospital, Yemetu, Ibadan, Nigeria |
| Campbell Ibijoke | Adeoyo Maternity Hospital, Yemetu, Ibadan, Nigeria |
| Adedapo B Ande | University of Benin Teaching Hospital, Benin-City, Nigeria |
| Aniekan Abbasiatai | University of Uyo Teaching Hospital, Uyo, Nigeria |
| Eno Etim Nyong | University of Uyo Teaching Hospital, Uyo, Nigeria |
| Sunny Ochigbo | University of Calabar Teaching Hospital, Calabar, Nigeria |
| Lawrence Omo‐Aghoja | Delta State University Teaching Hospital, Abraka, Nigeria |
| Patrick Ekpebe | Delta State University Teaching Hospital, Abraka, Nigeria |
| Anthonia Njoku | Federal Teaching Hospital, Irrua, Nigeria |
| Andrew Eigbedion | Federal Teaching Hospital, Irrua, Nigeria |
| Ngozi Orazulike | University of Port Harcourt Teaching Hospital, Port-Harcourt, Nigeria. |
| Chioma Okechukwu | University of Port Harcourt Teaching Hospital, Port-Harcourt, Nigeria. |
| Solomon Igbaruma | St Philomena Catholic Hospital, Benin, Nigeria |
| Idemudia Ebe | St Philomena Catholic Hospital, Benin, Nigeria |
| Osahon Ede-Edokpolor | Faith Mediplex Hospital Benin, Nigeria |
| Amarabia Ibeawuchi | Faith Mediplex Hospital Benin, Nigeria |
| Isa Ayuba Ibrahim | Niger Delta University, Wilberforce Highland, Nigeria |
| Oyedeji O Adeyemi | Niger Delta University, Wilberforce Highland, Nigeria |
| Chukwuemeka C Mgbafulu | Federal Teaching Hospital Abakaliki, Abakaliki, Nigeria. |
| Onubogu C Ukamaka | Nnamdi Azikiwe University Teaching Hospital, Nnewi, Nigeria. |
| Ugwu Anayochukwu | Enugu State University Teaching Hospital, Enugu, Nigeria. |
| Uchenna Ekwochi | Enugu State University Teaching Hospital, Enugu, Nigeria. |
| Obinna-Njoku Chioma | Federal Medical Centre, Owerri, Nigeria |
| George Eleje | Nnamdi Azikiwe University Teaching Hospital, Nnewi, Nigeria. |
| Eziamaka P Ezenkwele | University of Nigeria Teaching Hospital, Enugu, Nigeria. |
| Ijeoma Obumneme-Anyim | University of Nigeria Teaching Hospital, Enugu, Nigeria |
| Nnabuike Ojiegbe | Federal Medical Centre, Umuahia, Nigeria |
| Nathan U Nwokeforo | Federal Medical Centre, Umuahia, Nigeria |
| Ifeanyichukwu Ezebialu | Chukwu Emeka Ojukwu Teaching Hospital, Amaku, Nigeria |
| Obiora Ejiofor | Chukwu Emeka Ojukwu Teaching Hospital, Amaku, Nigeria |

**APPENDIX II World Health Organization’s Quality, Equity, and Dignity Indicators and Definitions**

| **Indicator** | **Operational definition** | **Method of data collection in current study** |
| --- | --- | --- |
| Intra-hospital maternal mortality ratio § | Number of maternal deaths (all women admitted and managed in the hospital regardless where she gave birth) per 100,000 live births | The number of maternal deaths recorded by facilities among all women admitted. Number of live births across the network (regardless of where baby was born) |
| Pre-discharge maternal mortality ratio | Number of deaths to women who delivered in the hospital per 100,000 hospital live births | The number of maternal deaths recorded by facilities among women who gave birth in the facility. Number of live births in the facility. |
| Institutional stillbirth rate (disaggregated by antenatal and intrapartum stillbirth) | Percentage of babies born in a health facility with no signs of life at birth, expressed as stillbirths per 1,000 facility births | The number of stillbirths (deaths ≥28 weeks) recorded by facilities among babies (≥28 weeks) who were born in the facility. |
| Pre-discharge neonatal mortality rate | Percentage of babies born alive in facility who died before discharge, expressed as deaths per 1,000 facility live-births | The number of babies (≥28 weeks) who died recorded by facilities among babies born alive in the facility. |
| Pre-discharge family planning counselling for mother and baby | Proportion of women who received pre-discharge family planning counselling for the mother and the baby in a given period | Number of women (obstetric admissions) being discharged with a live infant and received pre-discharge family planning counselling as recorded in postnatal ward note. In the case report forms, three responses where available: Yes (postnatal ward note reported that the woman had received pre-discharge family planning), No (postnatal ward note reported that the woman did not receive pre-discharge family planning), Information not available (it could not be ascertained from postnatal ward note if the woman received family planning counselling or not). |
| Companion of choice | The proportion of women who had a companion supporting them during labour and childbirth in the health facility | Number of women (obstetric admissions) who had a companion present as recorded in medical records. In the case report forms, three responses where available: Yes (medical record reported that the woman had a companion during labour), No (medical record reported that the woman did not have a companion during labour), unknown (medical record did not report if the woman had a companion in labour or not). If this information was left blank in the case report form it was considered missing data.  For women who had a companion in a labour the type of companion was also recorded (spouse, family member, or other person). |
| Newborns breastfed within one hour of birth | Percentage of babies born alive in a facility who are breastfed within one hour of birth | Number of babies born alive and were breastfed within one hour of birth as recorded in medical record. In the case report forms, three responses where available: Yes (medical record reported that baby was breastfed within one hour of birth), No (medical record reported that baby was not breastfed within one hour of birth), Information not available (it could not be ascertained from medical record if baby was breastfed within one hour of birth). |
| Immediate postpartum uterotonic use for postpartum haemorrhage (PPH) prevention | Percentage of women who gave birth in a facility who received a prophylactic uterotonic immediately after birth (ideally within one minute) for prevention of PPH | Number of women (obstetric admissions) who received a uterotonic for PPH prevention as recorded in medical records. In the case report forms, three responses where available: Yes (medical record reported that the woman received a uterotonic), No (medical record reported that the woman did not receive a uterotonic), unknown (medical record did not report if the woman received a uterotonic or not). If this information was left blank in the case report form it was considered missing data. |
| Newborns with birthweight documented | Percentage of babies born in a facility in a given period with documented birthweight before discharge | Number of babies born in facility with birthweight recorded in the medical record. In the case report form if birthweight (grams) was entered this was counted as a newborn who had birthweight documented. In case report forms where birthweight was left blank, this was counted as a newborn who did not have birthweight documented. |
| Basic Hygiene Provision | Proportion of facilities in which delivery rooms have at least one functional handwashing station with water and soap available | Number of facilities reporting having at least one functional handwashing station with water and soap available in most recent quarterly facility audit |
| Basic sanitation available to women and families | Proportion of facilities with basic sanitation available for women during and after labour and childbirth | Number of facilities reporting having basic sanitation available during and after labour and childbirth in most recent quarterly facility audit |

§ not a QED indicator but definition provided to assist interpretation of results presented in manuscript

**Appendix III. Maternal morbidity classifications (adapted from Say et al. 2009)**

***Woman without complication (healthy):*** no condition or morbidity recorded for the woman. i.e. health pregnancy with no conditions developed during pregnancy, no complications during childbirth or before discharge home.

***Woman with any complication:*** any morbidity recorded for the woman of any severity during pregnancy, childbirth or before discharge home, including any of the following:

1. **Obstetric complications (conditions that do not meet criteria for potentially life threatening)**: pre-existing condition (hypertension, diabetes, asthma, sickle cell anaemia, tuberculosis, HIV/AIDS, hepatitis, cardiac disease, renal disease, thyroid disease, epilepsy, other), gestational diabetes, placenta praevia, abruptio placenta, preterm labour, prolonged rupture of fetal membranes, anaemia, malaria, urinary tract infection, tuberculosis , HIV/AIDS, hepatitis, cardiac condition, renal condition, haemorrhage (blood loss >500mL<1000ml), pre-eclampsia, , retained placenta, wound infection, perineal tear (2^nd^ 3^rd^ or 4^th^ degree, high blood pressure (>140/90), induction or elective CS required for intrauterine growth restriction, intrauterine fetal death (induction of labour due to), congenital anomaly(detected antenatally) ; elective or emergency caesarean section (due to previous caesarean sections, cephalopelvic disproportion, cervical dystocia, prolonged labour, cord prolapse, oligohydramnios/anhydramnios, obstructed labour, breech presentation, multiple pregnancy, macrosomia , contracted pelvis, transverse lie, previous myomectomy, infertility, IVF baby, elderly primigravida, previous pelvic surgery, fetal distress, failed induction of labour, failed assisted vaginal birth, failed vaginal birth after caesarean section, failure of labour progress); assisted vaginal delivery (due to fetal distress, maternal exhaustion, delayed second stage, sickle cell disease, asthma);
2. **Gynaecologial complications (conditions that do not meet the criteria for potentially life threatening):** spontaneous miscarriage, induced miscarriage, molar pregnancy, missed abortion/fetal death (<28 weeks), high blood pressure (>140/90 mmHg),
3. any **potentially life-threatening condition (obstetric):** Woman has a serious condition which has the potential to be life threatening**:** ruptured uterus, eclampsia, severe postpartum haemorrhage (blood loss >=1000mL), puerperal sepsis, thromboembolic disease, acute renal failure, disseminated intravascular coagulopathy, stroke, anesthetic complication, pulmonary edema, cardiac arrest, hepatic failure; or admission to ICU for any reason.
4. any **potentially life-threatening condition (gynaecological):** haemorrhage, ectopic pregnancy, shock, acute renal failure, sepsis, thromboembolic disease, severe anaemia, organ perforation/injury, peritonitis, pelvic abscess, disseminated intravascular coagulopathy, blood transfusion or admission to ICU
5. **death:** death due to any cause

***Women with potentially life-threatening condition***: c and d above

**Appendix IV. Definitions of** **avoidable factors contributing to maternal or perinatal deaths from Nigerian National Maternal and Perinatal Death Audit Tool**

**Delay in woman seeking help**: describes the situation in which a woman either because of lack of awareness of pregnancy complications or owing to social reasons did not seek medical attention in time, as adjudged by the attending medical personnel.

**Lack/delay in transportation from home to healthcare facility:** delay in arrival at the hospital because of transportation problems, after a decision to seek medical care has been made.

**Delay in appropriate referral:** situation in which a hospital delayed in referring a woman to another hospital where she could have received definitive, life-saving treatment/procedure.

**Patient’s refusal of treatment or admission:** situation where the woman or her relative refuse life-saving intervention because of social, cultural or religious reasons. For example, the refusal of blood transfusion by a Jehovah’s Witness with severe postpartum haemorrhage.

**Delay in receiving care from medical staff**: situation where delay in instituting definitive treatment is primarily the fault of the medical personnel. For instance, on-call doctor sleeping at home which is more than 1km away.

**Lack of expertise, training or education:** situation where there is no medical staff knowledgeable enough to make appropriate diagnosis or provide the needed treatment.

**Lack of facilities, equipment or consumables**: situation where non-availability of equipment to perform certain life-saving procedure(s), lack of essential drugs or consumables hindered good quality care for the woman. For example, lack of anticonvulsant (e.g. magnesium sulphate) for the management of an eclamptic patient.

**Lack of human resources**: situation in which no medical record officers to open case file for the woman, no laboratory scientist to do blood test, no porter to wheel her to ultrasound room/theatre, no ambulance driver, etc. contributed to woman’s death.

**Health services communication breakdown:** situation in which lack of/gap in communication among health workers contributed to woman’s death. For instance, laboratory staff failed to alert the attending doctor about a high potassium result.
